# A BAC based physical map and genome survey of the rice false smut fungus *Villosiclava virens*

**DOI:** 10.1186/1471-2164-14-883

**Published:** 2013-12-16

**Authors:** Xiaoming Wang, Qingli Liu, Hao Wang, Chao-Xi Luo, Gejiao Wang, Meizhong Luo

**Affiliations:** 1 State Key Laboratory of Agricultural Microbiology, Huazhong Agricultural University, Wuhan, Hubei, 430070, PR China; 2 College of Life Sciences and Technology, Huazhong Agricultural University, Wuhan, Hubei, 430070, PR China; 3 College of Plant Science and Technology, Huazhong Agricultural University, Wuhan, Hubei, 430070, PR China

**Keywords:** Rice false smut, *Villosiclava virens*, BAC library, BAC end sequencing, BAC fingerprinting, Physical map

## Abstract

**Background:**

Rice false smut caused by *Villosiclava virens* is a devastating fungal disease that spreads in major rice-growing regions throughout the world. However, the genomic information for this fungal pathogen is limited and the pathogenic mechanism of this disease is still not clear. To facilitate genetic, molecular and genomic studies of this fungal pathogen, we constructed the first BAC-based physical map and performed the first genome survey for this species.

**Results:**

High molecular weight genomic DNA was isolated from young mycelia of the *Villosiclava virens* strain UV-8b and a high-quality, large-insert and deep-coverage Bacterial Artificial Chromosome (BAC) library was constructed with the restriction enzyme *Hin*dIII. The BAC library consisted of 5,760 clones, which covers 22.7-fold of the UV-8b genome, with an average insert size of 140 kb and an empty clone rate of lower than 1%. BAC fingerprinting generated successful fingerprints for 2,290 BAC clones. Using the fingerprints, a whole genome-wide BAC physical map was constructed that contained 194 contigs (2,035 clones) spanning 51.2 Mb in physical length. Bidirectional-end sequencing of 4,512 BAC clones generated 6,560 high quality BAC end sequences (BESs), with a total length of 3,030,658 bp, representing 8.54% of the genome sequence. Analysis of the BESs revealed general genome information, including 51.52% GC content, 22.51% repetitive sequences, 376.12/Mb simple sequence repeat (SSR) density and approximately 36.01% coding regions. Sequence comparisons to other available fungal genome sequences through BESs showed high similarities to *Metarhizium anisopliae, Trichoderma reesei, Nectria haematococca* and *Cordyceps militaris,* which were generally in agreement with the 18S rRNA gene analysis results.

**Conclusion:**

This study provides the first BAC-based physical map and genome information for the important rice fungal pathogen *Villosiclava virens*. The BAC clones, physical map and genome information will serve as fundamental resources to accelerate the genetic, molecular and genomic studies of this pathogen, including positional cloning, comparative genomic analysis and whole genome sequencing. The BAC library and physical map have been opened to researchers as public genomic resources (http://gresource.hzau.edu.cn/resource/resource.html).

## Background

Rice false smut caused by *Villosiclava virens* (Cooke Tak) (anamorph *Ustilaginoidea virens*) [1,2] has emerged as a devastating disease in rice, due to the intense application of nitrogen and phosphorus fertilizers and the cultivation of high-yielding semi-dwarf rice cultivars worldwide [3]. Previously, rice false smut was considered as a minor rice disease because of its rare occurrence in limited regions, but this disease has spread widely in the last 20 years and has become a severely devastating disease in many major rice-growing regions, including Asia, Africa, the United States, South America and Italy [3,4]. Rice false smut dramatically damaged rice production in 1988 and has continued to occur frequently [5]. The ustiloxin produced by this pathogen in infected plant tissues is a kind of cyclopeptide mycotoxins, which inhibits the growth of microtubules and is toxic to humans and livestock [6].

To date, the knowledge of *V. virens* is still very limited. Ashizawa *et al.* reported a sensitive method to quantify *V. virens* pathogens in soil samples using real-time PCR [4]. Ladhalakshmi *et al.* studied the intensity of rice false smut in India and found that the percentage of false smut-infected tillers ranged from 5% to 85% in the southern states, and 2% to 75% in northern states [7]. Atia *et al.* first investigated rice false smut in Egypt and reported that the production loss caused by this pathogen ranged from 1.0% to 10.9% [8]. Tanaka *et al.* established a simple transformation system of this pathogen using electroporation of intact conidial cells [2]. Fu *et al.* described the morphologic characteristics more precisely [3].

At present, the effect of rice false smut control is far from ideal. For searching the effective and environment-friendly methods, more morphological, molecular, genomic and genetic data of *V. virens* are required to reveal the infection process, the interaction mechanism between host and pathogen, the genetic variety and diversity, and the genome composition of this specie.

BAC libraries, physical maps and BESs serve as important tools in genetic, molecular and genomic studies. BAC libraries are used as templates in targeted or whole genome sequencing, physical map construction and functional complementation of genes in positional cloning. Physical maps provide frames for genome sequencing and physical positions of genes and markers. BESs are accurate and inexpensive genome samples [9], from which initial insights into the genome composition and candidates of molecular markers can be obtained [10,11]. The combined resources of BAC library, physical map and BESs of a genome play even more powerful roles synergistically in the above mentioned and extended research fields. BAC clones will largely increase the value and utility in targeted genome sequencing and positional cloning when mapped on a physical map. BESs embedded in physical map can be used as anchors in genome comparisons to detect sequence assembly errors of the same source genome and large structural changes of phylogenetically close genomes [12,13].

BAC libraries and physical maps have been constructed for several agriculturally important fungal organisms, such as *Magnaporthe oryzae*[14,15], *Blumeria graminis*[16], *Fusarium graminearum*[17], *Cryptococcus neoformans*[18], *Trichoderma reesei*[19] and *Ustilago maydis*[20]. We recently constructed a BAC library for a *V. virens* strain, UV-2 [21]. The BAC library contains 10,368 clones and has an average insert size of 124.4 kb. However, no physical map was constructed and no BESs were produced with this BAC library. Here we report the construction of a BAC-based physical map and genome survey of the *V. virens* strain UV-8b. To our knowledge, this is the first physical map and genome sequence information developed for *V. virens*.

## Results

### Phylogenetic analysis of strain UV-8b

The *V. virens* strain UV-8b was a single spore isolated from *Japonica* rice Zhonghua 11. To analyze the phylogenetic relationship between this strain and other fungal pathogens, we sequenced its 18S rRNA gene and compared it with other fungal 18S rRNA gene sequences. The 18S rRNA gene sequence of UV-8b showed a 99% identity to those of other *V. virens* strains and the phylogenetic tree constructed with the NJ algorithm clustered UV-8b into *V. virens* clade (Additional file 1: Figure S1). The UV-8b strain is also related to the members of *Metarhizium, Trichoderma* and *Cordyceps* (about 98% identities among 18S rRNA gene sequences).

### BAC library construction, fingerprinting and contig assembly

To obtain basic genome resources for *V. virens*, we constructed a BAC library and a BAC-based physical map of the *V. virens* strain UV-8b. The BAC library consists of 5,760 clones arrayed in 15 384-well plates. Analysis of 180 random BAC clones showed that the library had an average insert size of 140 kb with a size range from 25 to 190 kb and an empty-vector rate of lower than 1% (Table 1; Additional file 2: Figure S2). The library was calculated to cover 22.7-fold of the UV-8b genome (based on a genome size of 35.5 Mb, Dr. Shaojie Li, personal communication).

**Table 1:** **Statistics of the BAC Library, fingerprints and BESs of *V. virens *** **strain UV-8b**

**Category**	**Value**
Clone number	5760
Empty rate	< 0.1%
Average insert size	140 kb
Genome coverage^**1**^	22.7X
Clones end-sequenced	4512
Clones with BESs	3722
Paired-end BESs	2838
Single-end BESs	884
Average BES length	462 bp
GC content	51.52%
Clones fingerprinted	2688
Clones with fingerprints data^**2**^	2290
Assembled into FPC contigs	2035
With paired-end BESs	1557
With single-end BESs	407
As singletons	255
With paired-end BESs	194
With single-end BESs	48
Average bands per clone^**3**^	124

^**1**^The genome size of *V. virens* was estimated as 35.5 Mb.

^**2**^The clones that contained 50–200 bands were imported into FPC for contig assembly.

^**3**^The total bands of all successfully fingerprinted clones were 283391.

We fingerprinted 2,688 BAC clones using five restriction enzymes (*Bam*HI, *Eco*RI, *Xba*I, *Xho*I, *Hae*III). After quality filtering as described in the methods, fingerprint profiles of 2,290 BAC clones were qualified for FPC assembly. The 2,290 BAC clones covered 9-fold genome equivalents and contained an average of 124 bands (consensus bands; CBs) per clone. Based on the average insert size of 140 kb, one CB was estimated to be 1.13 kb (Table 1).

The fingerprint data of the 2,290 clones were imported into FPC V9.4 for contig assembly. A series of tests were performed to find optimal parameters for the assembly. Table 2 displayed the results of assembly with tolerance 4 and different cutoff values. Based on these tests, we chose 10^-15^ as the initial cutoff value for contig assembly. This condition setting assembled 2,035 clones into 196 contigs containing 111 (4.85%) Q clones, and left 255 (11.14%) clones as singletons. The contig101 (4 clones) was end-merged to contig76 (18 clones), and contig193 (29 clones) was end-merged to contig1 (3 clones), by the “End to End” function at terminal cutoff 10^-12^ and match value 2. This result was referred as PhaseIA and used as standard version. The PhaseIA contigs covered 51.2 Mb in physical length. The discrepancy between the genome length and the physical length of all contigs might be generated by the potential redundancy of contigs, which could be detected and merged with more evidences. Using “End to End” function at terminal cutoff 10^-08^ and match value 1, we merged another 74 contigs. This result was referred as PhaseIB. The BAC library and two versions of physical map have been opened to researchers as public genomic resources (http://gresource.hzau.edu.cn/resource/resource.html).

**Table 2:** Summary of the UV-8b physical maps autobuilt from assembly of the 2,290 BAC clones at different stringencies

**Cutoff**	**Contigs**	**Avr contig length (kb)**^**1**^	**Longest contig (kb)**^**1**^	**Physical length (Mb)**^**1**^	**Q contigs/ Q clones (%)**^**2**^	**No. contigs containing different clone numbers**					**Singletons (%)**^**3**^
						**>50**	**50-26**	**25-10**	**9-3**	**=2**	
10^-30^	314	184	287	58.2	0/0 (0.00)	0	0	18	180	116	991 (43.3)
10^-25^	305	196	376	60.0	7/7 (0.31)	0	0	43	168	94	653 (28.5)
10^-20^	255	217	774	55.5	36/49 (2.14)	0	4	57	136	58	428 (18.7)
10^-18^	231	230	774	53.4	41/63 (2.75)	0	9	61	112	49	357 (15.6)
10^-15^	196	261	774	51.3	63/111 (4.85)	1	15	61	78	41	255 (11.1)
10^-12^	161	302	1032	48.7	74/164 (7.16)	3	20	53	62	23	177 (7.7)
10^-10^	142	335	1585	47.7	77/174 (7.60)	5	20	51	48	18	160 (7.0)
10^-08^	135	340	1585	46.0	80/204 (8.91)	5	22	50	44	14	134 (5.9)
**Phase IA**^**4**^	194	263	774	51.2	63/111 (4.85)	1	15	61	76	41	255 (11.1)

^**1**^Each fingerprint band was estimated to be 1.13 kb based on the average insert size 140 kb and an average 124 bands per clone.

^**2**^The percentage of Q clones to total clones 2290.

^**3**^The percentage of single clones to total clones 2290.

^**4**^The cutoff value of 10^-15^ was chosen, then contig101was end-merged to contig76, and contig193 was end-merged to contig1 by “End to End” function at cutoff 10^-12^, respectively.

**Figure 1:**
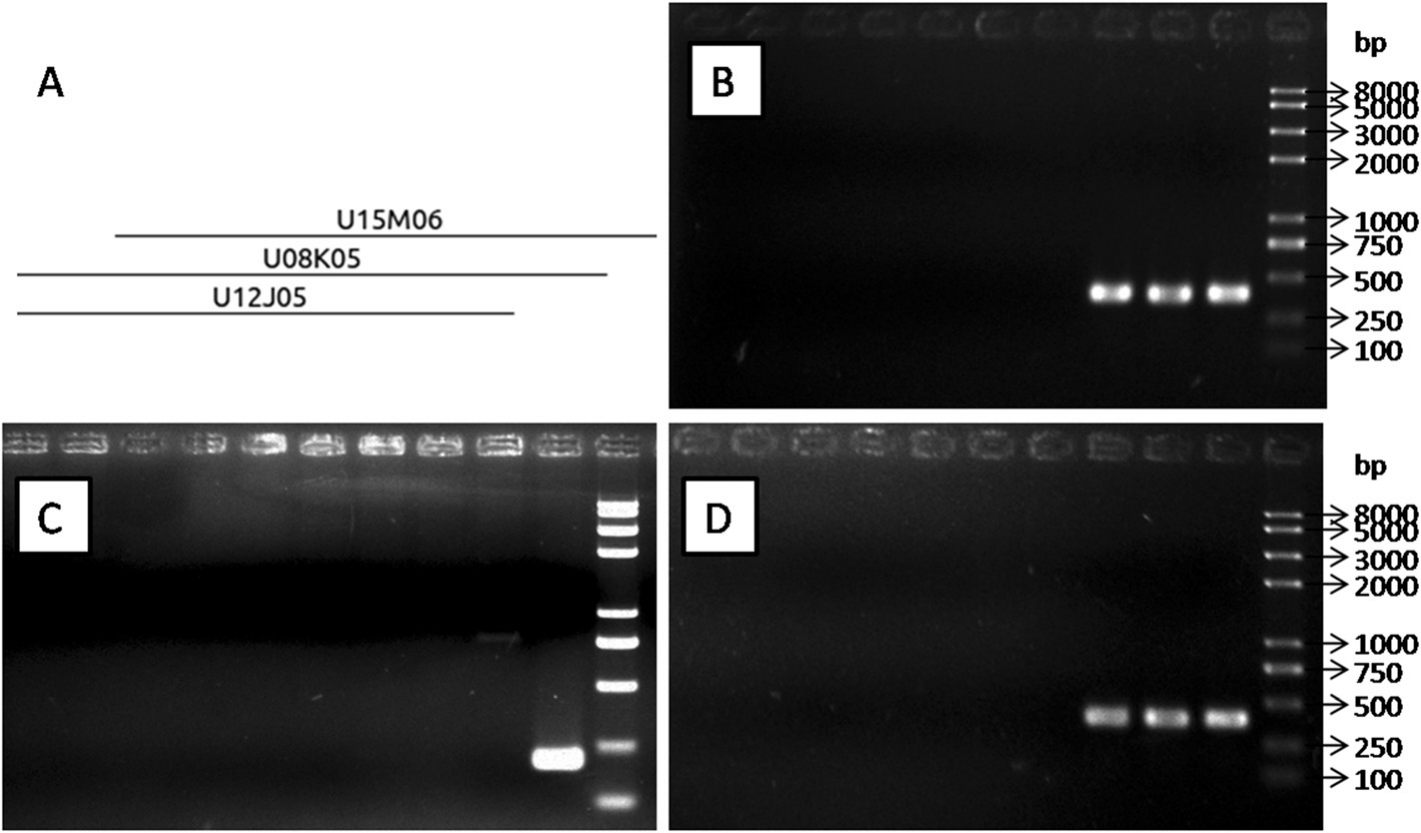
**Verification of the overlaps between clones in contig155.** Section **A**: Location of the clones in contig155; In section **B**, **C** and **D**, the templates used in PCR, from left to right, are: no template, host cells, empty vector, U13J12 (contig6), U03H01 (contig133), U03J10 (contig53), U03A03 (contig100), U12J05, U08K05 and U15M06. **B**: The pair primers were derived from BES of U12J05.f; **C**: The pair primers were derived from BES of U15M06.f; **D**: The pair primers were derived from BES of U15M06.r.

To evaluate the PhaseIA contig quality, we used PCR with primers from repeat masked BESs to verify the overlaps of clones in contigs. We randomly picked contig184 (2 clones), contig155 (3 clones), contig149 (4 clones), contig70 (7 clones), contig50 (12 clones), contig36 (20 clones) and contig17 (28 clones), and designed 3, 3, 4, 7, 8, 7 and 8 pairs of primers for PCR, respectively. As a result, only one clone (U08J21 located in the contig17) could not be distinctly confirmed (Figure 1; Additional file 3).

### BAC end sequencing

**Figure 2:**
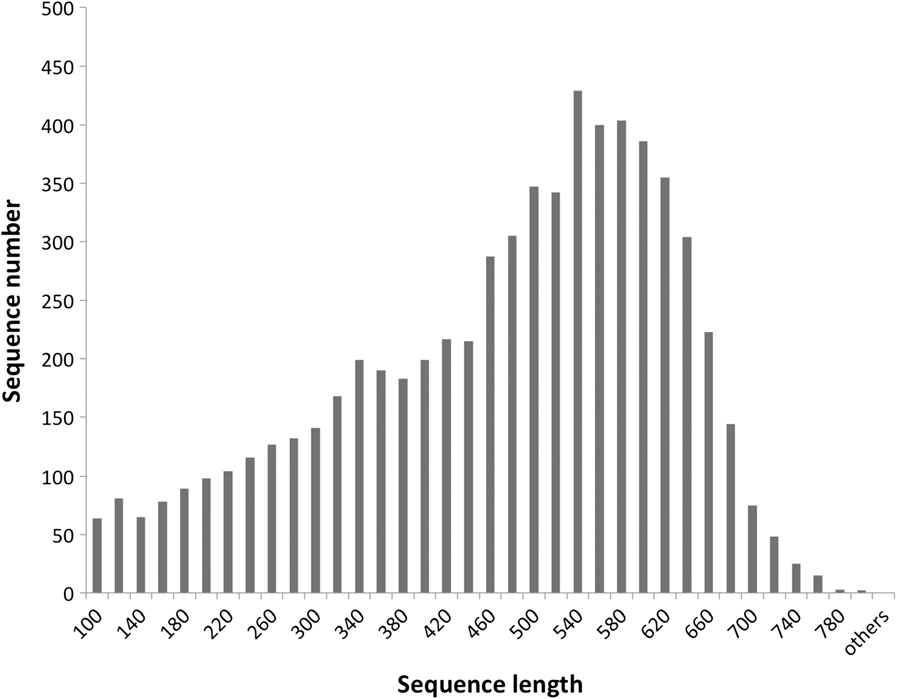
Distribution of BAC end sequence lengths.

To perform a genome survey and provide anchor sequences on the physical map for genome comparisons, we sequenced 4,512 BAC clones that included those clones used in fingerprinting at both ends. A total of 6,560 high quality BESs were generated after quality trimming, of which 5,676 were paired-end (86.52%) sequences and 884 were single-end sequences (13.48%) (Table 1). The maximal and the average length of the BESs were 798 bp and 462 bp (Figure 2), respectively. The total length of the BESs was 3,030,658 bp representing 8.54% of the whole genome. The GC content was 51.52%. The 6,560 high quality BESs are available in GenBank [GenBank:JY267549 to GenBank:JY274108].

### Analysis of repetitive DNA in BESs

Repeat sequences are usually a major component of eukaryotic genomes. To gain an initial insight into the composition of repeat elements contained in UV-8b BESs, RepeatMasker was used to identify the known repeat elements from existing databases. The result indicated that a total length of 138,502 bp (4.57%) of the known repeat sequences was identified and contained in 1,273 (19.41%) reads, among which only one read was completely recognized as repeat sequence. In the terms of the repeat category, retroelements were dominant and represented 3.07% of the total BES length, of which the LTR elements Ty1/Copia and Gypsy/DIRS1 accounted for 2.05% and 1.01%, respectively, while the LINE elements accounted for only 0.01% of the total BES length. Small RNA and simple repeats accounted for 0.04% and 0.95% of the total BES length, respectively (Table 3). It is interesting that few DNA transposons were identified in BESs in contrast to retroelements.

**Table 3:** Composition of known repetitive sequence types in the UV-8b BESs

**Type**	**Number of elements**	**Length occupied (bp)**	**Sequence (%)**^**1**^
Retroelements	314	93126	3.07
LINEs	3	306	0.01
LTR elements	311	92820	3.06
Ty1/Copia	208	62218	2.05
Gypsy/DIRS1	103	30602	1.01
DNA transposons	1	43	0.00
Hobo-activator	1	43	0.00
Small RNA	5	1199	0.04
Simple repeats	723	28868	0.95
Low complexity	366	15336	0.51

^**1**^The percentage of the length of each type to the total length of UV-8b BESs.

RepeatScout was used to *de novo* scan the repeat sequences contained in UV-8b BESs with the criterion described in the methods. A cumulative 682,351 bp (22.51%) were marked as repeat sequences with this pipeline, and were contained in 2,642 (40.27%) reads. Among these reads, 163 (2.48%) were marked as complete repeat sequences. The 1,384 reads in this result were not contained in the RepeatMasker result and 15 reads in the RepeatMasker result were not contained in this result. After repeat-masked, the BESs were self-BLASTed as described in the methods and no reads showed more than three matches to others, proving the high sensitivity of the RepeatScout pipeline.

### Comparative analysis of UV-8b with other fungal pathogens through BESs

For functional genomics comparison and evolutionary studies of the UV-8b genome, the following 10 well-characterized fungal pathogen genomes were chosen: *Magnaporthe oryzae, Botrytis cinerea, Puccinia* spp*, Fusarium graminearum, Fusarium oxysporum, Blumeria graminis, Mycosphaerella graminicola, Colletotrichum* spp*, Ustilago maydis* and *Melampsora lini.* They were voted as the most scientifically/economically important fungal pathogens by plant mycologists [22]. Four other fungi, *Metarhizium anisopliae, Trichoderma reesei, Nectria haematococca* and *Cordyceps militaris,* were also chosen as related species for this study, because they were close to *V. virens* in evolution distance and their whole genome sequences were available.

**Figure 3:**
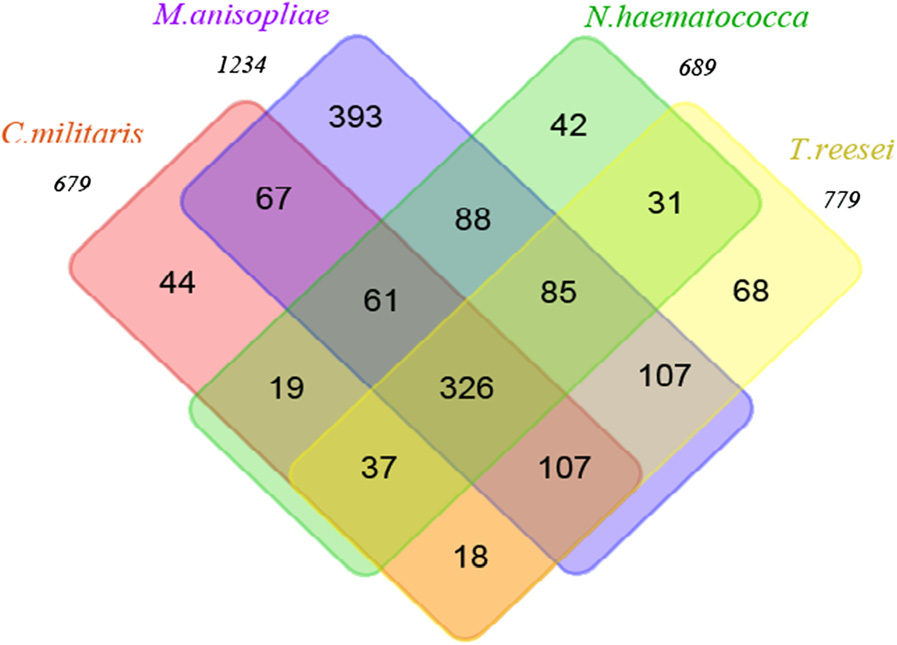
**The BLAST hit distribution of UV-8b repeat-masked BESs in four related fungal genomes.** The repeat-masked BESs were BLASTed against the four related fungal genomes using BLASTN program. A total of 1234, 779, 689 and 679 BESs hit to *M. anisopliae, T. reesei, N. haematococca* and *C. militaris* genome sequences, respectively. The shared BES hits are illustrated here; for example, 326 repeat-masked BESs hit to the four genomes simultaneously.

To identify the microsynteny regions of UV-8b to the above genomes, the repeat-masked UV-8b BESs were used in BLAST analysis with the above genome sequences. As shown in Table 4, 0.18-8.34% of masked BESs matched to the top 10 plant fungal pathogen genomes. In ascomycetes pathogens, *F. oxysporum* (8.34%) and *F. graminearum* (8.19%) showed the most hits, followed by *C. graminicola* (8.13%), and *B. graminis* (1.07%) showed the least hits. In basidiomycetes pathogens, *U. maydis* (0.95%) has the smallest genome size but the highest number of hits; *P. graminis* (0.18%) and *Melampsora laricis* (0.24%) showed less hits. Among masked BESs, 18.81%, 11.88%, 10.50% and 10.35% matched to *M. anisopliae, T. reesei, N. haematococca* and *C. militar*, respectively, and 326 masked BESs matched to all of those species (Figure 3). The similarity results were generally in agreement with the 18S rRNA gene analysis.

**Table 4:** Comparative analysis of UV-8b BESs with the top ten fungal pathogens and four related fungal genomes

**Fungus genome**			**No. of masked BESs with BLAST hits**^**1**^	**No. of BES pairs with BLAST hits**	**On the same chromosome, scaffold or contig**		**In correct orientation**^**2**^		**Within 50–500 kb**		**Correct orientation and within 50-500 kb**	
**Species**^**3**^	**Length (Mb)(GC content)**	**Scaffold or contig of more than 300 kb**^**4**^				**No. of mapping loci**		**No. of mapping loci**		**No. of mapping loci**		**No. of mapping loci**
*Magnaporthe oryzae*	41.73 (51.55%)	40.87 (97.94%)	344 (5.24%)	7	5	6	2	2	1	1	0	0
*Botrytis cinerea*	42.66 (43.07%)	18.00 (42.19%)	118 (1.80%)	0	0	0	0	0	0	0	0	0
*Puccinia graminis*	88.72 (43.35%)	72.98 (82.25%)	12 (0.18%)	0	0	0	0	0	0	0	0	0
*Fusarium graminearum*	36.55 (48.28%)	36.09 (98.74%)	537 (8.19%)	30	14	21	6	8	7	8	2	2
*Fusarium oxysporum*	61.44 (48.38%)	57.76 (94.01%)	547 (8.34%)	27	12	15	7	9	6	7	3	4
*Blumeria graminis*	128.76 (43.97%)	20.65 (16.04%)	70 (1.07%)	0	0	0	0	0	0	0	0	0
*Mycosphaerella graminicola*	39.69 (52.14%)	39.69 (100.0%)	154 (2.35%)	0	0	0	0	0	0	0	0	0
*Colletotrichum graminicola*	51.64 (49.11%)	38.53 (74.60%)	533 (8.13%)	32	8	11	5	7	4	5	1	1
*Ustilago maydis*	19.68 (54.03%)	2.03 (10.34%)	62 (0.95%)	1	0	0	0	0	0	0	0	0
*Melampsora laricis*	101.13 (41.0%)	90.04 (89.04%)	16 (0.24%)	0	0	0	0	0	0	0	0	0
*Metarhizium anisopliae*	39.15 (51.49%)	32.46 (82.93%)	1234 (18.81%)	121	48	63	32	36	27	34	20	24
*Trichoderma reesei*	33.40 (52.83%)	30.50 (91.33%)	779 (11.88%)	55	15	25	5	7	7	15	2	4
*Nectria haematococca*	51.29 (50.79%)	42.25 (82.38%)	689 (10.50%)	41	19	27	8	11	13	18	5	7
*Cordyceps militaris*	32.27 (51.42%)	32.21 (99.82%)	679 (10.35%)	41	18	24	4	4	10	13	1	1

^**1**^Percentage relative to the total number of masked BESs (6,560).

^**2**^The two end sequences of the same clone hit to the “+” and “-” strand, respectively.

^**3**^Except that the *Puccinia graminis, Ustilago maydis* and *Melampsora laricis* were basidiomycetes, the other 7 of the top 10 pathogens all are ascomycetes.

^**4**^The accumulative length of scaffolds or contigs of more than 300 kb and percentage relative to the total length of assembled sequences.

Among the BLAST hits, if paired-ends hit to target genomes with the criteria described in [9], the regions were considered to be collinear between UV-8b and the target genomes. The results (Table 4) showed that *F. oxysporum* and *F. graminearum* have more collinear regions than the others in the top 10 pathogen genomes. In the four related genomes, an insect fungal pathogen *M. anisopliae* had the most collinear regions. The higher degree of synteny between UV-8b and *M. anisopliae* was consistent with the results of the species distribution in the gene annotation step. Since most of the target genomes were not assembled completely (Table 4), the numbers of paired-end BESs potentially collinear with target genomes could be higher than detected.

**Figure 4:**
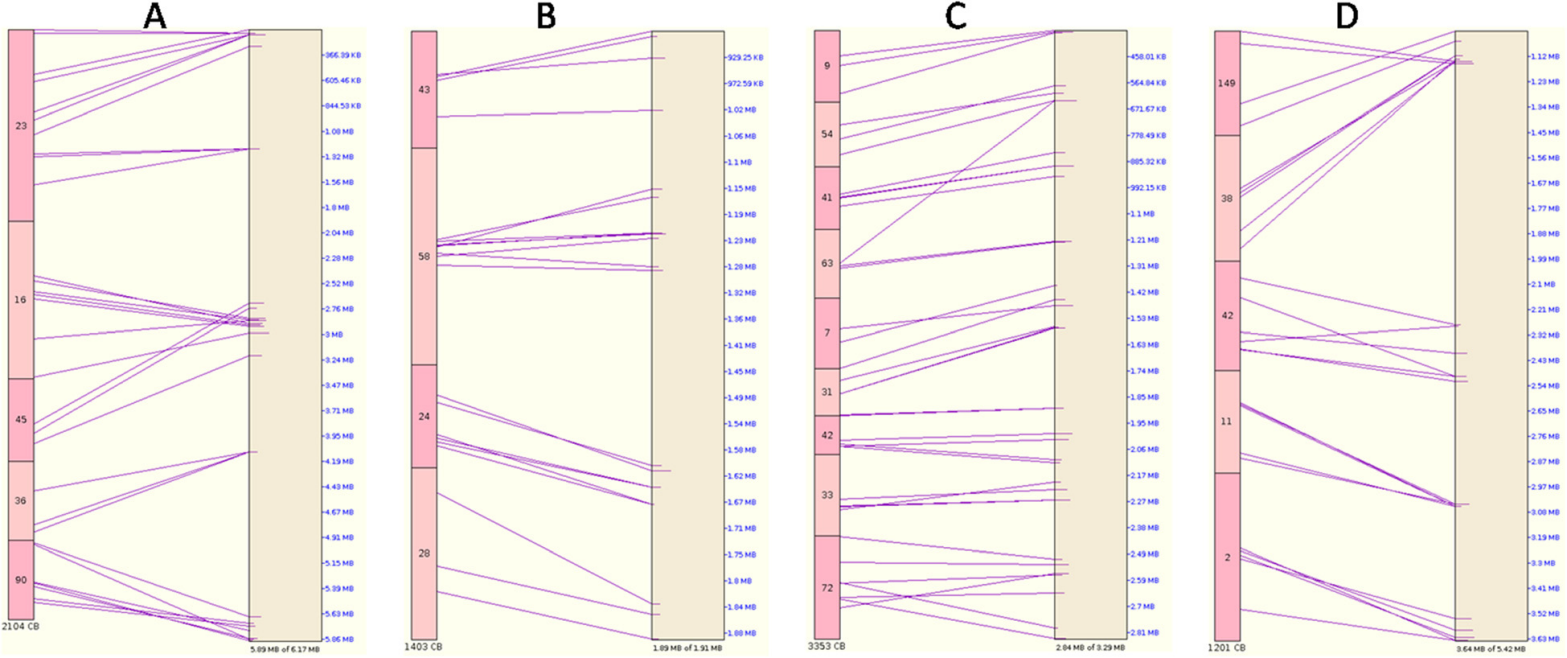
**Mapping UV-8b PhaseIA contigs to the four related genome sequences.** The UV-8b PhaseIA contigs were anchored to the four fungal genomes based on the repeat-masked BESs. The left bars represent UV-8b fingerprint contigs and the right boxes represent each of the four related genome sequences. The purple lines represent the BES alignments. **A**: Alignments between UV-8b contigs and the partial draft sequence of *M. anisopliae* (gi 322711353). **B**: Alignments between UV-8b contigs and the partial draft sequence of *T. reesei* (gi 340520780). **C**: Alignments between UV-8b contigs and the partial draft sequence of *N. haematococca* (gi 302917142). **D**: Alignments between UV-8b contigs and the partial draft sequence of *C. militaris* (gi 346324065).

In order to detect large syntenic regions, we used the SyMAP [23] program based on the BESs embedded in the contigs to anchor UV-8b PhaseIA contigs to the genomes of *M. anisopliae, T. reesei, N. haematococca* and *C. militaris*. Under the SyMAP default criteria, *M. anisopliae* had most anchored contigs, followed by *T. reesei* (Table 5), consistent with the comparative analysis results mentioned above (Table 4). Figure 4 shows an example of the graphical representation of the collinear regions.

**Table 5:** The summary of the SyMAP results

** Category**	***Metarhizium anisopliae***	***Trichoderma reesei***	***Nectria haematococca***	***Cordyceps militaris***
BESs with hits	551 (8%)	381 (5%)	332 (5%)	335 (5%)
In blocks^**1**^	300 (54%)	144 (37%)	130 (39%)	116 (34%)
Paired BESs^**2**^	18	4	5	5
Anchored contigs	60	35	30	29
Map coverage^**3**^	21%	12%	11%	11%

^**1**^The syntenic regions identified by SyMAP.

^**2**^The distance between the two hits of one clone in reference is within 500 kb.

^**3**^The CB percentage of the anchored contigs to total contigs.

### Analysis of simple sequence repeats (SSRs)

SSRs are potential genetic markers due to their high rate of polymorphisms. To investigate the SSR contents and their distribution in UV-8b BESs, we scanned the BES dataset with SciRoko3.4 [24]. First, the CAP3 [25] program was used to reduce the redundancy of BESs; it clustered 1,821 BESs into 803 contigs and left 4,739 reads as singletons. The total length of these reads was 2,719,880 bp. Among these random genome sequences, a total of 1,023 SSR loci were identified from 849 reads with the criterion described in the methods. The SSRs had an average length of 25.17 bp, an average standard deviation of 10.55 bp and a density of 376.12/Mb.

Of these SSRs, 339 (33.14%), 57 (5.57%), 223 (21.80%), 113 (11.05%), 158 (15.44%), and 133 (13.00%) represented mononucleotide, dinucleotide, trinucleotide, tetranucleotide, pentanucleotide, and hexanucleotide types, that were composed of 2, 3, 10, 23, 53 and 85 SSR motifs, respectively (Table 6). The most abundant SSR types are mononucleotide and trinucleotide, in which the most abundant SSR motifs are A (268) and AGC (42), respectively. The dinucleotide (29.40 bp) and hexanucleotide (29.28 bp) SSR types had the longest average lengths among the different SSR types. The AACC motif (42.33 bp) had the longest length among the different SSR motifs. With the SSRs and their flanking sequences as input, a total of 836 pairs primers were designed for SSR loci by the Primer3 [26] program. Information on the SSRs and the primers was showed in an additional file (Additional file 4: Table S1).

**Table 6:** The SSR frequency and distribution in the UV-8b BESs

**Type (motif number)**^**1**^	**Average length (bp)**	**SSR number**	**Counts/Mb**	**Read number**^**2**^	**Primer number**^**3**^
Mononucleotide (2)	22.68	339	124.64	329	248
A	23.76	268	98.53	258	204
C	18.59	71	26.10	71	44
Dinucleotide (3)	29.40	57	20.96	57	44
AG	28.48	33	12.13	33	23
AT	30.27	15	5.51	15	14
AC	31.33	9	3.31	9	7
Trinucleotide (10)	27.16	223	81.99	215	191
AGC	29.98	42	15.44	37	37
AAG	31.63	40	14.71	38	33
ACG	27.00	36	13.24	36	32
CCG	22.40	30	11.03	30	27
AAT	17.75	16	5.88	16	13
ATC	31.38	16	5.88	16	13
others	25.33	43	15.81	42	36
Tetranucleotide (23)	25.14	113	41.55	113	100
AAAG	25.46	13	4.78	13	12
AGGC	24.92	12	4.41	12	10
ACCT	23.27	11	4.04	11	11
AGCC	30.33	9	3.31	9	6
others	25.84	68	25.01	68	61
Pentanucleotide (53)	22.72	158	58.09	155	137
AAAAG	25.82	22	8.09	20	15
AAAAT	21.06	16	5.88	16	14
ACCAG	27.00	10	3.68	10	10
others	22.02	110	40.44	109	98
Hexanucleotide (85)	29.28	133	48.90	133	116
AAAAAG	33.67	6	2.21	6	5
AGCGGC	24.00	5	1.84	5	4
ATATCG	20.40	5	1.84	5	5
others	32.13	117	43.01	117	102
**Total**	25.17	1023	376.12	849	836

^**1**^Group all the similar and reverse complementary SSR motifs together, e.g. “TC”, “CT”, “AG”, and “GA” were grouped into “AG”.

^**2**^The number of sequence reads that contain SSRs.

^**3**^The primer numbers that were successfully designed for SSR loci.

**Figure 5:**
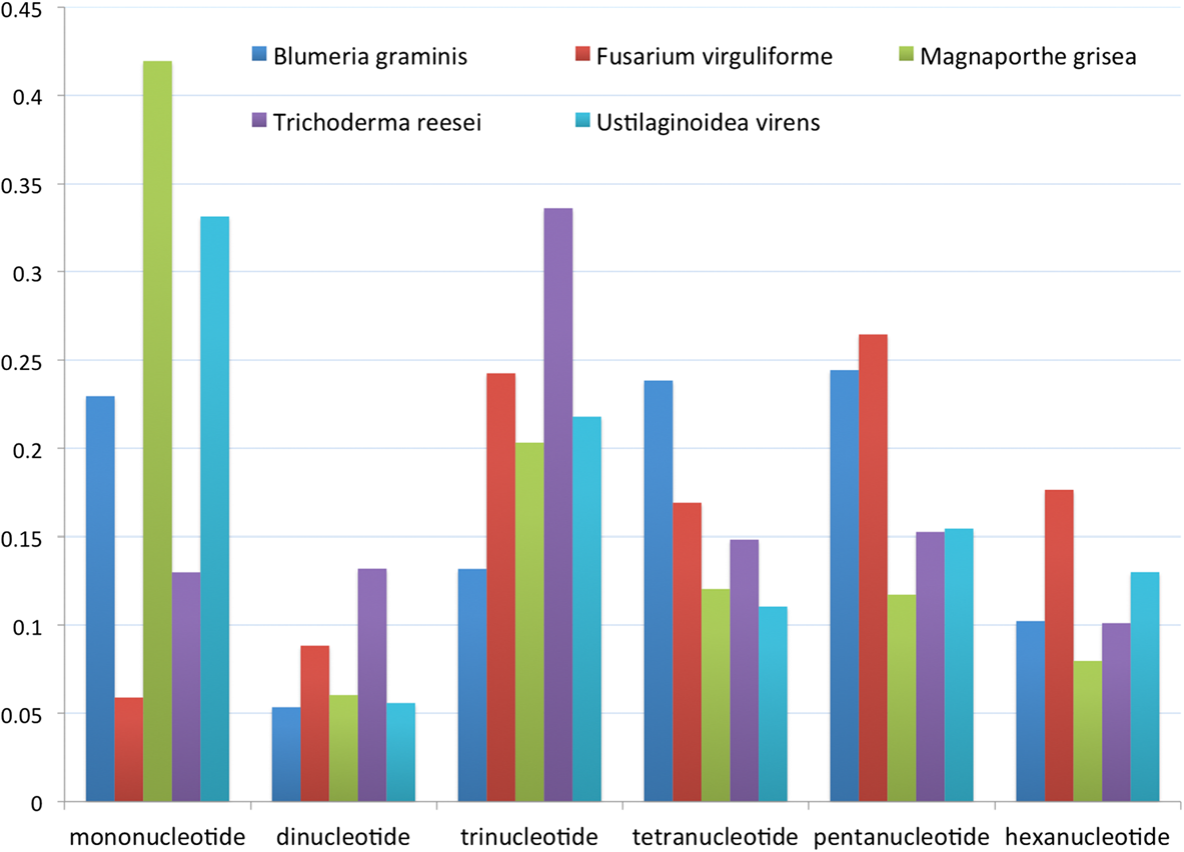
**The SSR content and distribution in different fungal genomes.** The GSS sequence data of *B. graminis, F. virguliforme, M. oryzae* and *T. reesei* as well as UV-8b BESs were scanned for SSRs. The Y-axis represents the percentage of each SSR type to the total SSRs.

To compare the SSR contents and distribution patterns, the GSS sequences of *B. graminis, Fusarium virguliforme, M. oryzae* and *T. reesei* were downloaded from NCBI and scanned for SSRs using the same parameters of CAP3 and SciRoKo programs. The SSR contents and distribution patterns varied obviously (Figure 5). The SSR densities of the above species were 96.27/Mb, 107.71/Mb, 173.60/Mb and 234.06/Mb, respectively, in contrast to 376.12/Mb in UV-8b. The result indicated that UV-8b and *T. reesei*, which were closest in phylogenetic distance among the four species, had the highest SSR densities. The frequencies of the SSR types were also different among the above species. Mononucleotide types were dominant in UV-8b and *M. oryzae*, whereas the trinucleotide was most common in *T. reesei*. It is interesting that dinucleotides had the lowest frequency in all of the species. As for the frequencies of individual SSR motifs, SSR motif A was the most common motif in UV-8b, *M. oryzae* and *B. graminis*, AG was most common in *T. reesei*, and AGC was most common in *F. virguliforme*.

### Gene annotation

**Figure 6:**
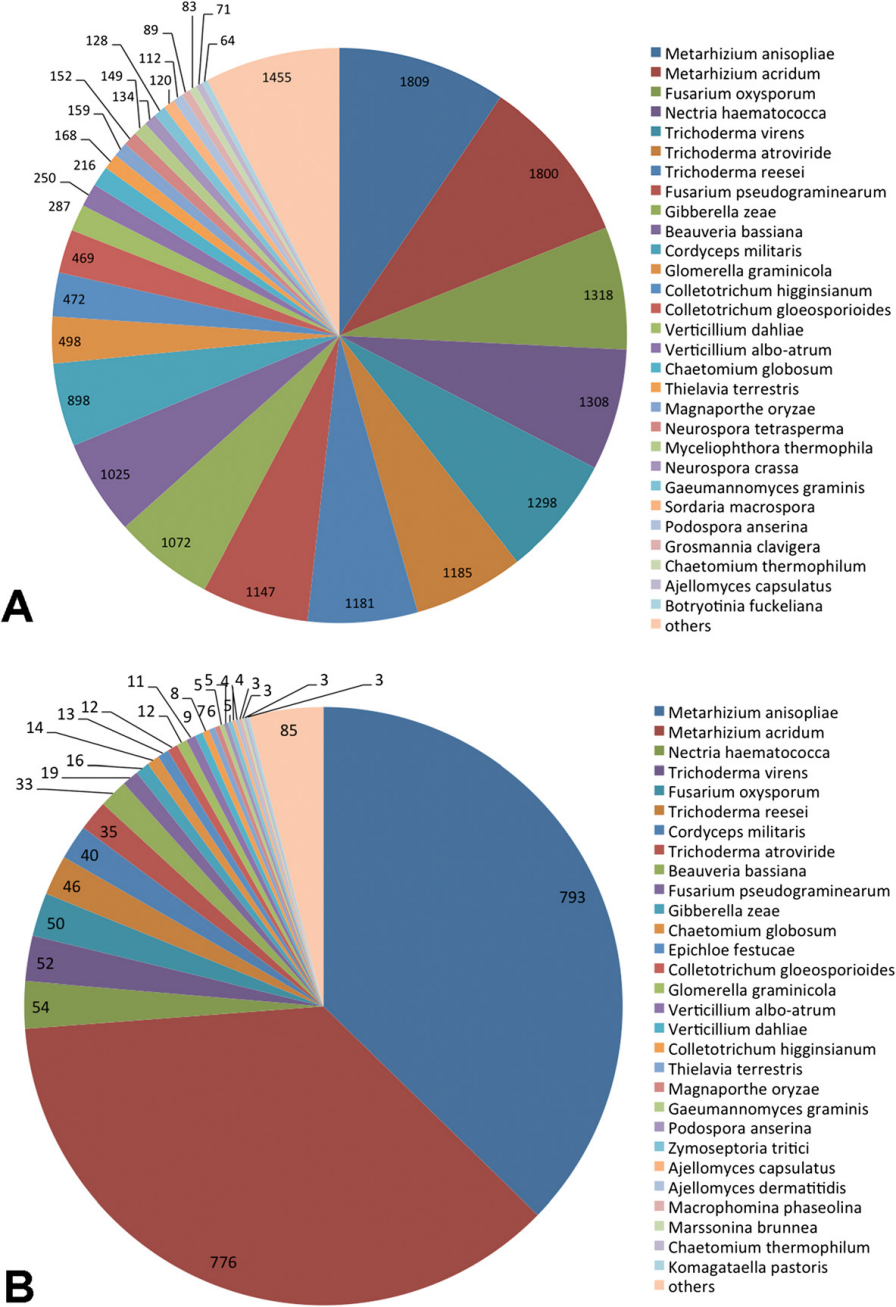
**Distribution of the matched species in annotation step.** The masked and non-redundant BESs were BLASTed against the NR database of NCBI using BLASTX program. **A**: All-Hit species distribution. All BLAST hits of the BESs were counted. **B**: Top-Hit species distribution. Only the best BLAST hit was counted.

Before gene annotation, the repeat-masked BESs were pre-processed by the CAP3 [25] program to reduce sequence redundancy. A total of 640 contigs were formed by the CAP3 program and 5,215 reads were left as singletons. The cumulative length of the processed sequences was 2,797,772 bp. An additional 876 (398,742 bp) reads, whose effective lengths were shorter than 100 bp, were removed to improve the result accuracy. The final 4,979 (contigs + singletons) reads, whose total length was 2,399,048 bp, were compared with the EST and NR databases of NCBI to identify coding regions. A total of 1,592 (31.97%) reads with a cumulative length of 835,095 bp (34.81%) were identified as homologous to ESTs (E-value cutoff of ≤ 10^-10^). Found to match to NR database (E-value cutoff of ≤ 10^-6^), were 2,219 (44.57%) reads with a cumulative length of 1,149,495 bp (47.91%), of which 1,492 reads were homologous to both the EST and the NR database. Taken together, 2,319 (46.58%) reads (1,592 + 2,219-1,492) with a cumulative length of 1,194,135 bp (49.78%) were identified to contain coding regions, and the cumulative length of coding regions was 863,826 bp, representing 36.01% of the total sequences (2,399,048 bp). Figure 6 shows the target species distribution in the NR database. *M. anisopliae* and *M. acridum* had the most BLASTX hits.

**Figure 7:**
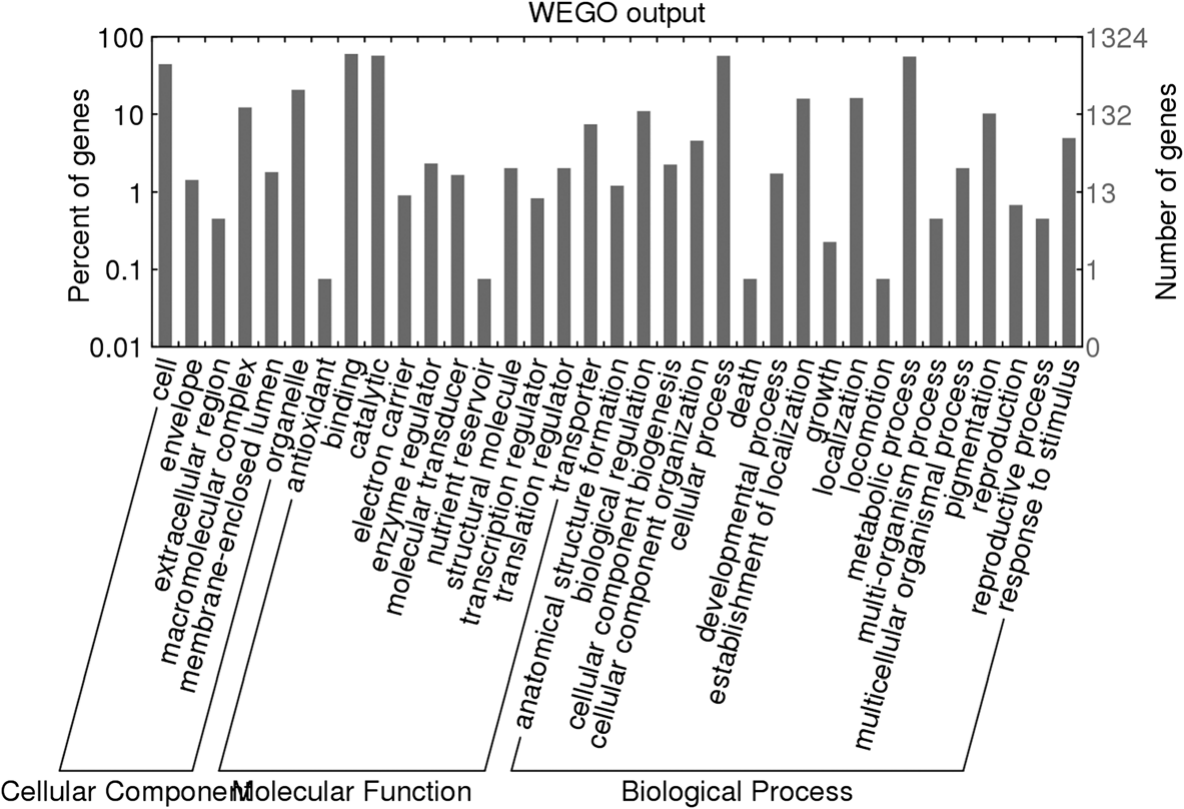
Distribution of GO annotations of gene products predicted from UV-8b BESs.

A total of 928 unique GO terms were assigned to 1,324 reads, and each read was associated with 3.37 GO numbers on average. The genes showed a wide range of functional categories (Figure 7; Additional file 5: Table S2). The binding and catalytic activities were most abundant in the molecular function category, whereas the cellular and metabolic processes were most common in the biological process category. A total of 971 reads matched to the InterProScan database provided a reliable dataset to understand gene function. On the other hand, 171 unique EC (Enzyme Code) annotations were assigned to 387 reads, and 74 pathways in which these enzymes participated were identified by the KEGG map module of BLAST2GO [27], such as the tricarboxylic acid cycle (TCA cycle). Six enzymes (from 7 reads) of the 171 unique EC were involved in the TCA cycle.

## Discussion

Rice is the staple food of more than 50% of people worldwide, and the problem of food deficiency is more and more severe with the expanding human population [28,29]. Rice false smut caused by *V. virens* has emerged as a devastating disease in rice, and the ustiloxin produced by the pathogen is toxic to humans and livestock [6]. However, little is known about this fungal pathogen to date. In this study, we constructed the first BAC-based physical map and generated a large set of BESs for *V. virens*. These resources will serve as fundamental tools for molecular, genetic and genomic studies of this pathogen.

Due to the lack of reference sequences and effective molecular markers, the contigs could not be edited. We used PCR with the primers derived from the masked BESs to evaluate the contig quality. From a total of 76 clones analyzed, only one clone was not verified by the PCR experiment, indicating that the contig assembly is reliable. The control samples and several pairs of primers in one contig helped to discriminate the false positive PCR bands.

Transposable elements (TEs) contribute largely to the evolution of fungal genomes [30,31]. In UV-8b, we found that the known repeat elements represented 4.57% of the total BESs and are mainly LTR elements. Few DNA transposons were identified. This may be because the percentage of retroelements is higher than DNA transposons in the *V. virens* repeat family or because DNA transposons of *V. virens* are less homologous with the available repetitive sequences in the Fungi sub-database of RepeatMasker. In the *M. oryzae* genome, the retroelements were also more common than DNA transposons [32,33].

By *de novo* searching the repetitive sequences contained in the UV-8b BES dataset with RepeatScout, a total of 682,351 bp (22.51%) sequences distributed in 2,642 reads (40.27%), were marked as repeat sequences. In our results, the core-repetitive sequences that were identified by RepeatScout were identified in 4 to 156 BESs, while the fragments which have lower occurrence may be false positives or lowly repetitive sequences. However, the percentage of repeat sequences reduced from 22.51% to 16.25% if the criterion threshold for hits in BESs was set as >5 instead of >3 times (please note that the BES sequences accounted for only 8.54% of the genome sequence). There was 10.3% genome sequences that were identified as repetitive sequences in the *M. oryzae* p131 assembly [32].

A total of 1,384 reads identified by RepeatScout were not identified by RepeatMasker, indicating that they are new repeat elements that have not been collected in the database. Fifteen known repeat element-containing reads identified by RepeatMasker were not identified by RepeatScout. It is possible that these elements have high-copy numbers in other fungi but low-copy numbers in the UV-8b genome or that they were under-represented in the BESs.

Bischoff *et al*. analyzed the phylogenetic placement of *Villosiclavae* and claimed that it is related to, but distinct from, the *Clavicipitaceae* and *Hypocreaceae* clades [34]. This is in agreement with our phylogenetic analysis that most of the strains closely related to *V. virens* UV-8b belong to *Clavicipitaceae* and *Hypocreaceae*. In the processes of both genome comparison (Figure 3; Table 4) and gene annotation (Figure 6), the BLAST hit distributions were also consistent with the result of the phylogenetic tree, except for with *T. reesei*. This result could be due to the fact that the genome sequences used for genome comparison were draft sequences, and less genomic resources of *T. reesei* were deposited in GenBank for gene annotation.

To date, little is known about co-linearity of chromosome segments among filamentous ascomycete fungi [33] compared to plant and animal genomes. The synteny relationship could facilitate the acquisition of knowledge about genome evolution and dynamics, comparative genomics and phylogeny [35,36]. We compared the repeat-masked BESs to the top 10 fungal pathogens to search microsyntenic regions. The result showed that *F. oxysporum* and *F. graminearum* have the most hit numbers. The hosts of *F. oxysporum* range from arthropods [37] to humans and also include gymnosperm and angiosperm plants [38], whereas *F. graminearum* was notorious for causing *Fusarium* head blight. However, the *M. oryzae*, which was a well-known pathogen of rice, showed few synteny regions.

Alignments of contigs and BAC clones to the target or reference genomes through BESs were widely used to detect phylogenetic relationships and large structural genomic variations between species, such as expansion, contraction, inversion and rearrangement in plants [12,39]. These alignments could also assist in the sequence assembly and detect the assembly errors of the genome sequence of the same species [13]. In this study, the numbers of UV-8b contigs aligned to the target genomes were not high. This was most probably due to both the high diversities among fungal genomes and the incompleteness of the target genome sequences.

The SSRs play an important role in genetic diversity analysis and genetic map construction due to their high level of polymorphisms, co-dominance and robustness [40]. Before substantial genome sequence availability, the BESs, as random genome survey sequences, were an important resource for mining SSR markers. We found 1,023 SSRs with an average length of 25.17 bp and a density of 376.12/Mb, of which primers of 836 loci have been designed successfully. These primers are candidates for genetic analysis by PCR. It is interesting that UV-8b has a similar SSR content and distribution pattern with *M. oryzae* but not with the closely related *T. reesei.*

## Conclusions

We constructed the first generation BAC-based physical map of *V. virens* and acquired 3,030,658 bp of BAC end sequences, representing 8.54% of the genome. The BAC library was equivalent to 22.7X genome coverage with an average insert size of 140 kb. A total of 2,035 BAC clones were assembled into 194 contigs and 255 clones were left as singletons. The BAC library and physical map provide tools for positional cloning, comparative genomics and whole genome sequencing of *V. virens*. In addition, the BAC end sequence analysis provides a glimpse into the *V.virens* genome composition, such as 51.52% GC content, 22.51% repetitive sequences, 376.12/Mb SSR density and approximately 36.01% coding regions. We believe that all these information is valuable to expedite the genomic and genetic research into the important rice false smut fungus.

## Methods

### The 18S rRNA gene identification and phylogenetic analysis

The 18S rRNA gene of UV-8b was amplified by PCR using the common primers NS1 and NS8 [41]. The sequence was compared with those in the NCBI GenBank database by the BLASTN searching tool. The sequences were edited using ClustalX 1.83 software [42]. The phylogenetic tree was constructed using the neighbor-joining (NJ) algorithm tested by 1,000 bootstrap with MEGA5 [43].

### High molecular weight genomic DNA preparation

The *V. virens* strain UV-8b was subcultured on PSA medium (1 L: 200 g peeled potato, 20 g sucrose and 15 g agar; natural pH) at 28°C for 5 days. The fresh mycelium was harvested and transferred onto new PSA plates covered with a layer of cellophane to propagate enough amount of fresh mycelium. The fresh mycelium was collected, ground properly and cultured in liquid complete medium [44,45] at 28°C, 180 rpm for 65 h. The culture was filtered through 2–4 layers of cheese cloth. The collected mycelium pellet was washed first with sterile ddH_2_O twice and then with 0.7 M NaCl twice, and incubated in 0.7 M NaCl solution containing 8 mg/ml Driselase (SIGMA D9515) at 31°C at 100 rpm for 3 h to release protoplasts. The protoplast-containing mixture was filtered through one layer of miracloth twice. The protoplast-containing solution was centrifuged at 1500 g for 15 min. The pellet was washed with 0.7 M NaCl for three times, with 1.2 M sorbitol once and then resuspended in a minimal volume (usually ~1 ml) of 1.2 M sorbitol to reach a compromise to obtain both as high as DNA concentration and as many as DNA plugs required for at least one attempt of BAC library construction (It is difficult to obtain a high DNA concentration from rice fungi). The protoplast suspension was mixed with an equal volume of 1% low melting point (LMP) agarose (prepared with 1.2 M sorbitol) at 45°C and then transferred into plug molds (Bio-Rad) to form plugs. The plugs were treated following our published protocol [46].

### BAC library construction

BAC library construction was performed as previously described [46-48]. The linearized dephosphorylated low-copy BAC vector pIndigoBAC536-S was prepared with *Hin*dIII from a high-copy composite vector pHZAUBAC1 as previously described in Shi *et al.*[49]. Individual BAC clones were arrayed in 384-well plates and stored at −80°C in our laboratory. The insert size of the BAC library was estimated by digesting random BAC clones with I-*Sce*I and analyzing the digested products on 1% CHEF agarose gel at 5–15 s linear ramp, 6 V/cm, 14°C in 0.5× TBE buffer for 17 h.

### BAC plasmid DNA preparation

BAC plasmid DNA was extracted as described by Kim *et al.*[39] with minor modifications. BAC clones were inoculated in deep 96-well plates with a 96-well replicator, and each well contained 1.2 ml of 2 × YT medium plus 12.5 μg/ml chloramphenicol. The plates were covered with Airpore gas-permeable plate sealant (AXYGEN) and shaken on an orbital shaker at 180 rpm at 37°C for 20 hours. BAC plasmid DNA was extracted manually using the AxyPrep™ Easy-96 Plasmid Kit (24×96-prep (AXYGEN)), according to manufacturer’s instructions, and dissolved in 35 μl 1 mM Tris–HCl, pH 8.0.

### BAC fingerprinting and contig assembly

BAC fingerprinting was performed using SNaPshot kit (ABI No. 4323159) as described by Luo *et al.*[50] and Kim *et al.*[39] with minor modifications. SNaPshot reaction products were purified and dissolved in 10 μl of Hi-Di formamide (ABI NO. 4311320) containing 0.15 μl of GeneScan-500 LIZ Size Standard (ABI No. 4322682). An ABI 3730 DNA analyzer with 50 cm capillaries (Applied Biosystems, Foster City, California) was used to separate fingerprinting fragments. Fingerprint profiles that contained fragment peaks between 50 and 200 were collected. FPC software version V9.4 [51] was used for contig assemblies. The FPC parameters were adjusted as described [50,52]. A series of cutoff and tolerance values were tested to obtain optimal assembly following the principles of decreasing the number of contigs without excessively increasing the number of questionable clones. After each round, when more than 5 Q clones existed in a contig, the “DQer” function was used to break up the Q contig with a step value of 2. Finally, the tolerance value was set to 4 and the Sulston cutoff value was set to 10^-15^. At the end, the contigs were improved using the “End to End” automerge function.

The primers which were used to evaluate the contig quality generated at cutoff 10^-15^ were designed from masked BESs by primer5, with the exception of contig184 whose 3 pairs of primers were SSR primers generated in SSR analysis. The conditions of the bacterial liquid PCR reaction were 94°C for 5 min for initial denaturation, followed by 35 cycles of denaturation at 94°C for 30 sec, annealing for 30 sec, and extension at 72°C for 40 sec, and a final cycle of extension for 10 min. The annealing temperature was selected based on the TM values of the primers. The products of PCR were separated in 1.0% agarose gels. The presence/absence of the bands of expected sizes were examined. The host cells, empty vector, and the clones U13J12 (contig6), U03H01 (contig133), U03J10 (contig53), and U03A03 (contig100) were randomly selected as control samples.

### BAC end sequencing

BAC end sequencing was performed as previously described [53] with some modifications. BAC clones were sequenced at both ends on an ABI 3730 DNA Analyzer using Big-Dye v3.1 (Applied Biosystems, Foster City, California), following the manufacturer’s instructions. The two primers BACf (5′aacgacggccagtgaattg3′) and BACr (5′gataacaatttcacacagg3′) were used as forward and reverse sequencing primers, respectively. Sequences were base-called using Phred [54], and the vector and low-quality (Phred value <16) sequences were removed using the program LUCY [55]. The reads less than 100 bp in length were removed. All the trimmed sequences were deposited in the GenBank database [GenBank:JY267549 to GenBank:JY274108].

### Analysis of repetitive DNA in BESs

The known classes of repeat elements contained in the UV-8b BAC end sequences were identified by the RepeatMasker v3.3.0 pipeline (http://www.repeatmasker.org) from the Fungi subdatabase in RepBase17.07 [56]. The BAC end sequences were used to search for novel repeat elements with RepeatScout1.0.5 [57]. Only the sequences that were repeated > 3 times and were > 50 bp in length in the BES dataset were kept. Then, the remaining BES sequences were self-BLASTed to search for additional BESs that were repeated > 3 times and were > 50 bp in length [12,58].

### Genome comparative analysis

The genome sequences of the fungal pathogens *M. oryzae, B. cinerea, P. graminis, F. graminearum, F. oxysporum, C. graminicola* and *U. maydis* were downloaded from the Broad Institute Database (http://www.broadinstitute.org). The genome sequences of the fungal pathogens *B. graminis, M. graminicola, M. laricis, M. anisopliae, N. haematococca, T.reesei* and *C. militaris* were downloaded from the NCBI database (http://www.ncbi.nlm.nih.gov). The repeat-masked BESs were BLASTed against the above genome sequences using BLASTN with an E-value cutoff of 10^-05^. The matched sequences with longer than 50 bp and more than 80% identity were collected and analyzed. The BESs were also used to anchor the corresponding contigs to the genome sequences of *M. anisopliae, F. oxysporum, N. haematococca* and *T. reesei* using the SyMAP V3.4 program [23] (http://www.agcol.arizona.edu/software/symap/).

### Analysis of simple sequence repeats

The BES sequences were clustered by the program CAP3 [25] with default parameters to reduce the redundancy of the dataset. The non-redundant sequences were scanned by SciRoko3.4 [24] to search for the potential SSRs, with the criteria of a minimum repeat number was of 3 and a minimum total length of 15 bp. The full standardization of SciRoKo, which groups all the similar and complementary SSR motifs together, was used for the SSR statistics, e.g. “TC”, “CT”, “AG”, and “GA” were grouped into “AG”. The genome sequences (GSS section) of *B. graminis, F. virguliforme, M. oryzae* and *T. reesei* were downloaded from NCBI (of December 2012) and mined for SSRs with the same criteria above for comparisons of the SSR contents and distribution patterns. The primers flanking SSRs were designed by standalone primer3 [26] and the DesignPrimers program in the SciRoko3.4 package [24].

### Gene annotation

After the repeat elements were masked, the masked-BESs were clustered by CAP3 [25] with default parameters to reduce redundancy. To identify protein-coding regions, the masked and non-redundant BESs were BLASTed to the GenBank EST database using the program BLASTN with an E-value cutoff of 10^-10^ and to the non-redundant protein database using the program BLASTX with an E-value cutoff of 10^-06^. Different functions of the program BLAST2GO [27] were used to analyze the BLASTX-identified sequences: GO terms were annotated by the GO function, the motifs and domains were identified by the InterProScan function and the pathways were annotated by the Enzyme Code and KEGG function.

## Competing interests

The authors declare that they have no competing interests.

## Authors’ contributions

XW carried out the BAC end sequencing, fingerprinting, contig quality evaluation, data analysis and elaborated this manuscript. QL constructed this BAC library and carried out the BAC end sequencing, fingerprinting and the phylogenetic analysis. HW carried out the BAC end sequencing and fingerprinting. ML, GW and C-XL designed the project. ML supervised this work. ML and GW finalized this manuscript. All the authors read and approved the final manuscript.

## Supplementary Material

Additional file 1: Figure S1 Neighbor-joining phylogenetic tree of 18S rRNA gene sequences. Phylogenetic tree showing the phylogenetic position of the strain UV-8b and the strains of the genera *Villosiclava*, *Trichoderma*, *Metarhizium, Cordyceps* and *Torrubiella*. Percentages at nodes are bootstrap values based on 1,000 replications. The scale bar represents 0.01 substitutions per nucleotide position. The 18S rRNA gene sequences of the strains UV-8b and UV-2 were generated in our laboratory. The 18S rRNA gene sequence of *T. reesei* QM6a was obtained by the draft genome sequence in BLAST. The 18S rRNA gene sequences of other strains were obtained from GenBank. Click here for file

Additional file 2: Figure S2 Insert size analysis of randomly selected UV-8b BAC clones. The plasmid DNA of 180 randomly selected BAC clones from the UV-8b BAC library were digested with I-*Sce*I, and the DNA fragments were separated on 1% CHEF agarose gel. Lane 21 was the MidRange PFG marker I (NEB). V is vector. Click here for file

Additional file 3 **Contig quality evaluation.** The primers have been designed from repeat sequence masked BAC end sequences and were used to verify the overlap between clones in PhaseIA contig. Click here for file

Additional file 4: Table S1 Information about the SSRs and the primers. With the SSRs and their flanking sequences as input, a total of 836 pairs primers were designed for SSR loci by Primer3 program. Click here for file

Additional file 5: Table S2 GO annotations of gene products predicted from UV-8b BESs. GO annotations and terms at level 3. Click here for file
