# Supplementary figures and images for: A BAC based physical map and genome survey of the rice false smut fungus Villosiclava virens
Source: BMC Genomics. 2013 Dec 16;14:883. doi: 10.1186/1471-2164-14-883 (PMC3878662; doi:10.1186/1471-2164-14-883)

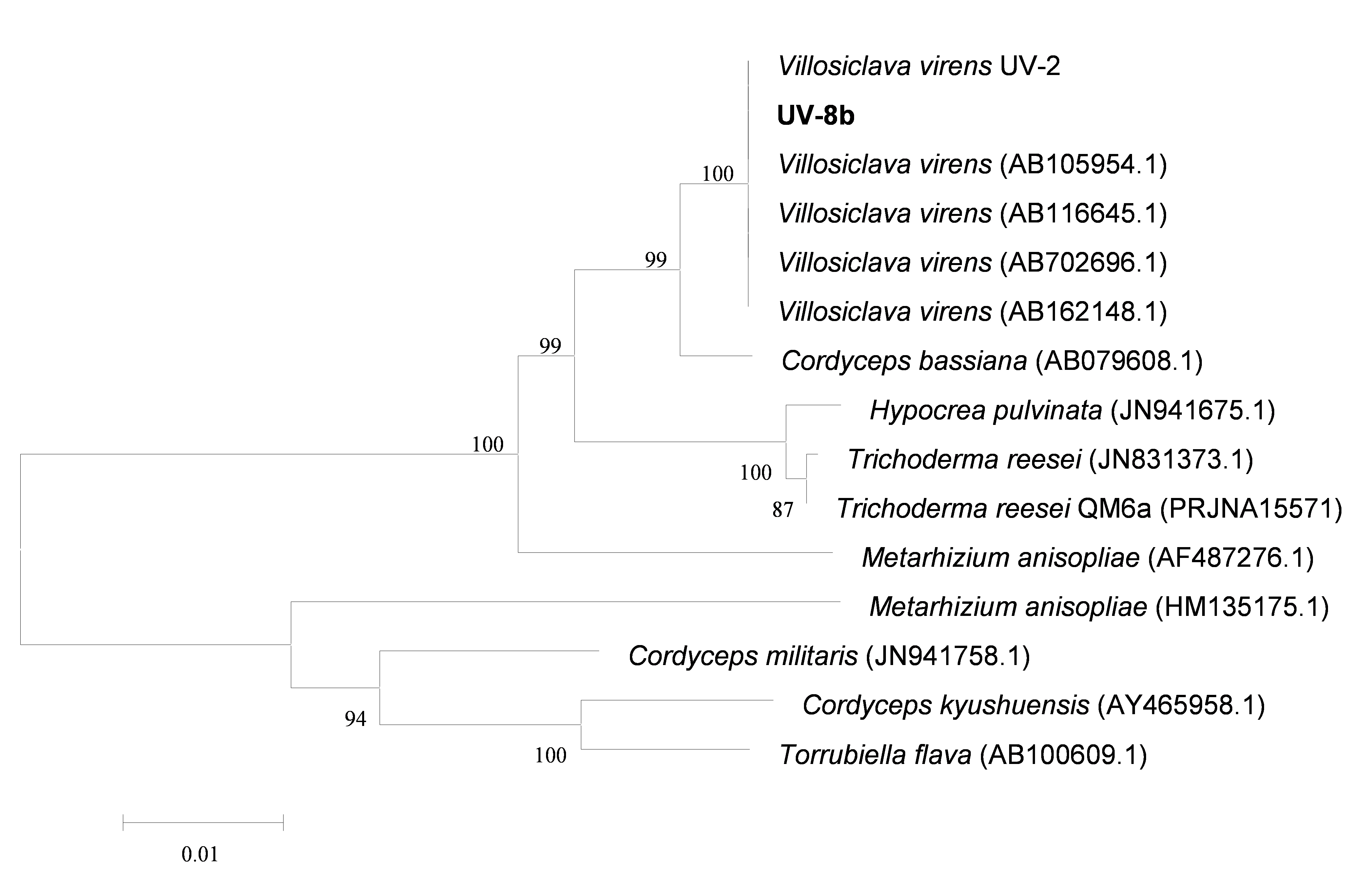

Supplement: Additional file 1: Figure S1 — Neighbor-joining phylogenetic tree of 18S rRNA gene sequences. Phylogenetic tree showing the phylogenetic position of the strain UV-8b and the strains of the genera Villosiclava, Trichoderma, Metarhizium, Cordyceps and Torrubiella. Percentages at nodes are bootstrap values based on 1,000 replications. The scale bar represents 0.01 substitutions per nucleotide position. The 18S rRNA gene sequences of the strains UV-8b and UV-2 were generated in our laboratory. The 18S rRNA gene sequence of T. reesei QM6a was obtained by the draft genome sequence in BLAST. The 18S rRNA gene sequences of other strains were obtained from GenBank. [file 1471-2164-14-883-S1.png]

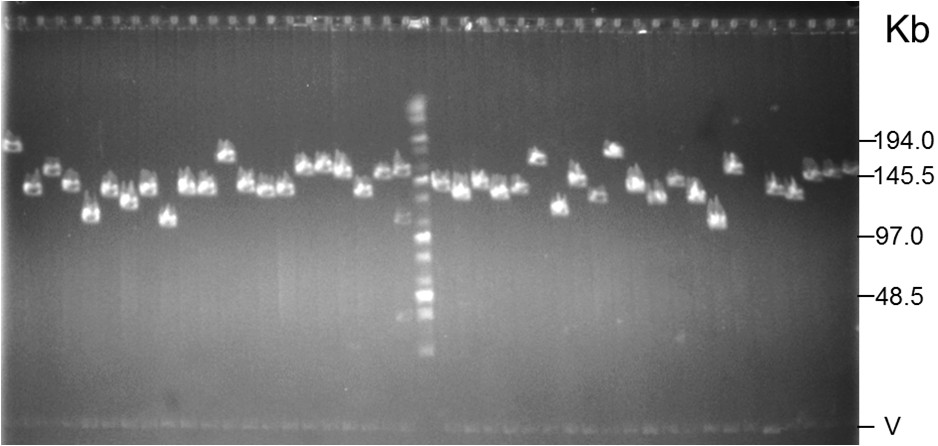

Supplement: Additional file 2: Figure S2 — Insert size analysis of randomly selected UV-8b BAC clones. The plasmid DNA of 180 randomly selected BAC clones from the UV-8b BAC library were digested with I-SceI, and the DNA fragments were separated on 1% CHEF agarose gel. Lane 21 was the MidRange PFG marker I (NEB). V is vector. [file 1471-2164-14-883-S2.png]
