# Supplementary material for: A BAC based physical map and genome survey of the rice false smut fungus Villosiclava virens
Source: BMC Genomics. 2013 Dec 16;14:883. doi: 10.1186/1471-2164-14-883 (PMC3878662; doi:10.1186/1471-2164-14-883)
Supplement: Additional file 3 — Contig quality evaluation. The primers have been designed from repeat sequence masked BAC end sequences and were used to verify the overlap between clones in PhaseIA contig. [file 1471-2164-14-883-S3.doc]

# Contig quality evaluation

Due to the lack of reference sequence and molecular markers for *Villosiclava virens*, we performed PCR using the primers derived from the repeat-masked BESs to evaluate the contig quality. Host cells, empty vector and clones in different contigs were used as control samples. It is worth to note that, in contigs, the clone length could not accurately reflect the real insert size because only the fingerprints, whose lengths were between the minimum and maximum values of the GeneScan-500 LIZ Size Standard (ABI No. 4322682), were calculated for each clone in fingerprinting. Also, a BES can be assigned to a clone but cannot be sure to which end of the clone.

The marker used in this experiment is Trans2K PlusII DNA Marker:


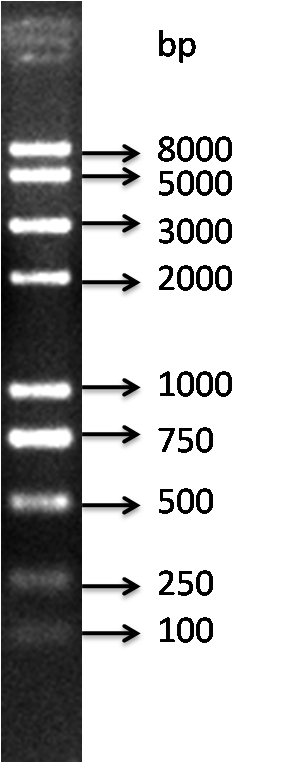


## A: Quality evaluation of contig184


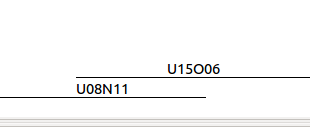


**Figure 1** **Location of the clones in contig184**


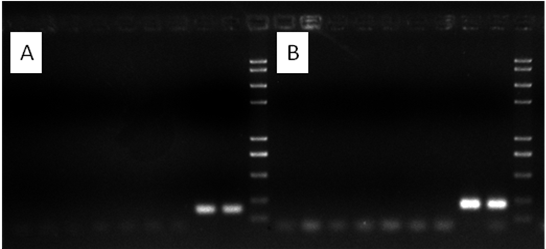

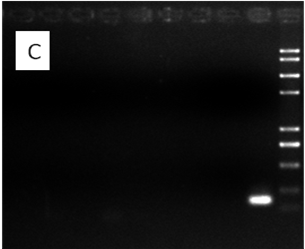


**Figure 2 Verification of the overlaps between clones in contig184**

In section A, B and C, the templates used in PCR, from left to right, are: no template, host cells, empty vector, U13J12 (contig6), U03H01 (contig133), U03J10 (contig53), U03A03 (contig100), U08N11 and U15O06. A: The pair primers were derived from BES of UO8N11.f; B: The pair primers were derived from BES of UO8N11.r; C: The pair primers were derived from BES of U15O06.r. These three pairs of primers were SSR primers and designed automatically in SSR analysis by primer3. This result implied that the lengths of one or both clones of this contig might be incorrectly calculated in fingerprinting and the clone UO8N11 might be contained in the clone U15O06.

## B: Quality evaluation of contig149


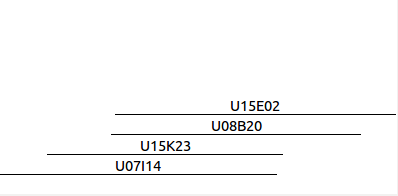


**Figure 1 Location of the clones in contig149**

**
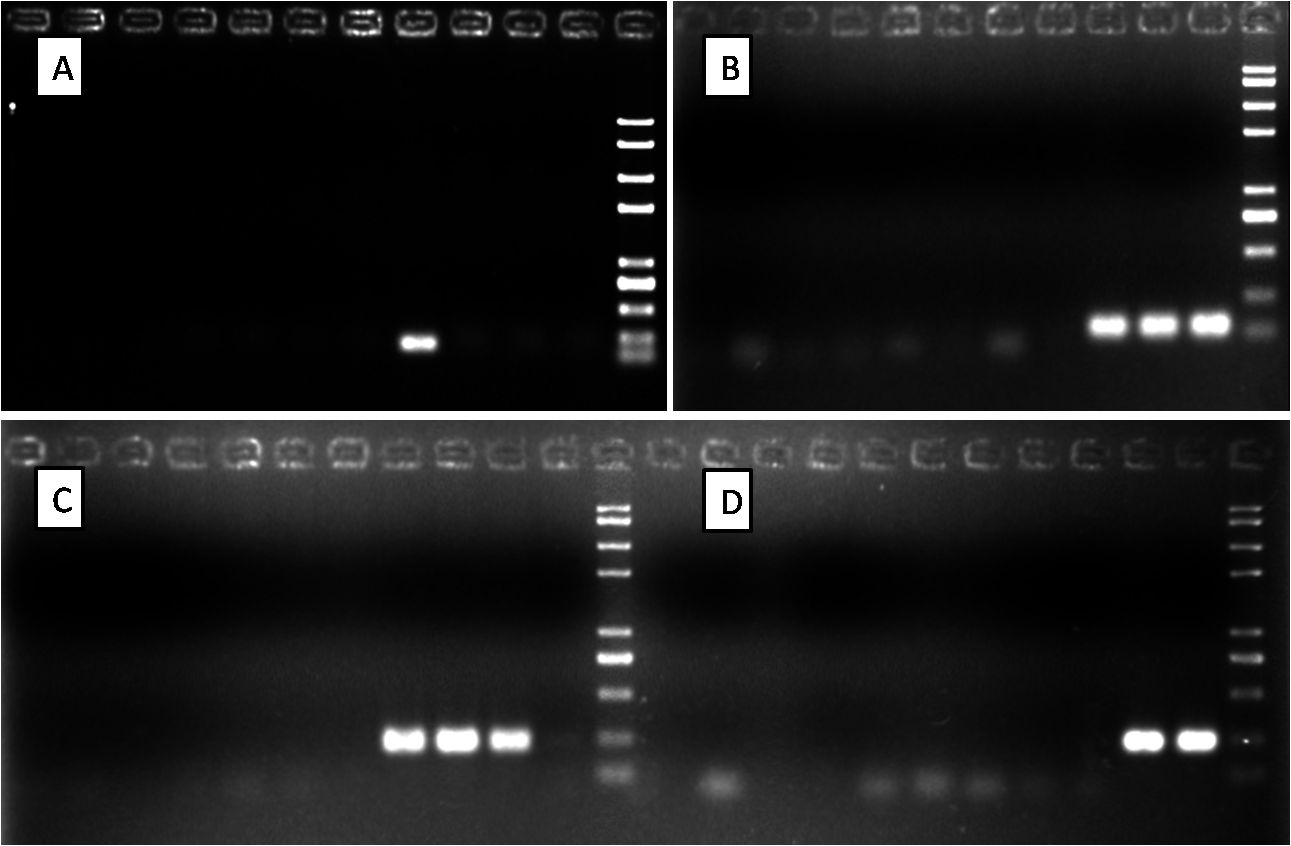
**

**Figure 2 Verification of the overlaps between clones in contig149**

In section A, B, C and D, the templates used in PCR, from left to right, are: no template, host cells, empty vector, U13J12 (contig6), U03H01 (contig133), U03J10 (contig53), U03A03 (contig100), U07I14, U15K23, U08B20, and U15E02. A: The pair primers were derived from BES of U07I14.f; B: The pair primers were derived from BES of U15K23.f; C: The pair primers were derived from BES of U08B20.f; D: The pair primers were derived from BES of U08B20.r.

## C: Quality evaluation of contig70


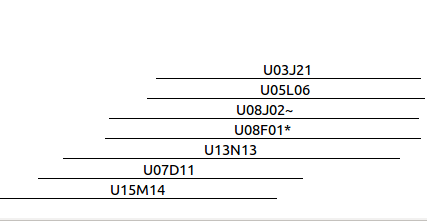


**Figure 1 Location of the clones in contig70**

* The clone U08F01 contains clone U08J02 in physical location in this contig.

**~** The clone U08J02 is contained by clone U08F01 in physical location in this contig.


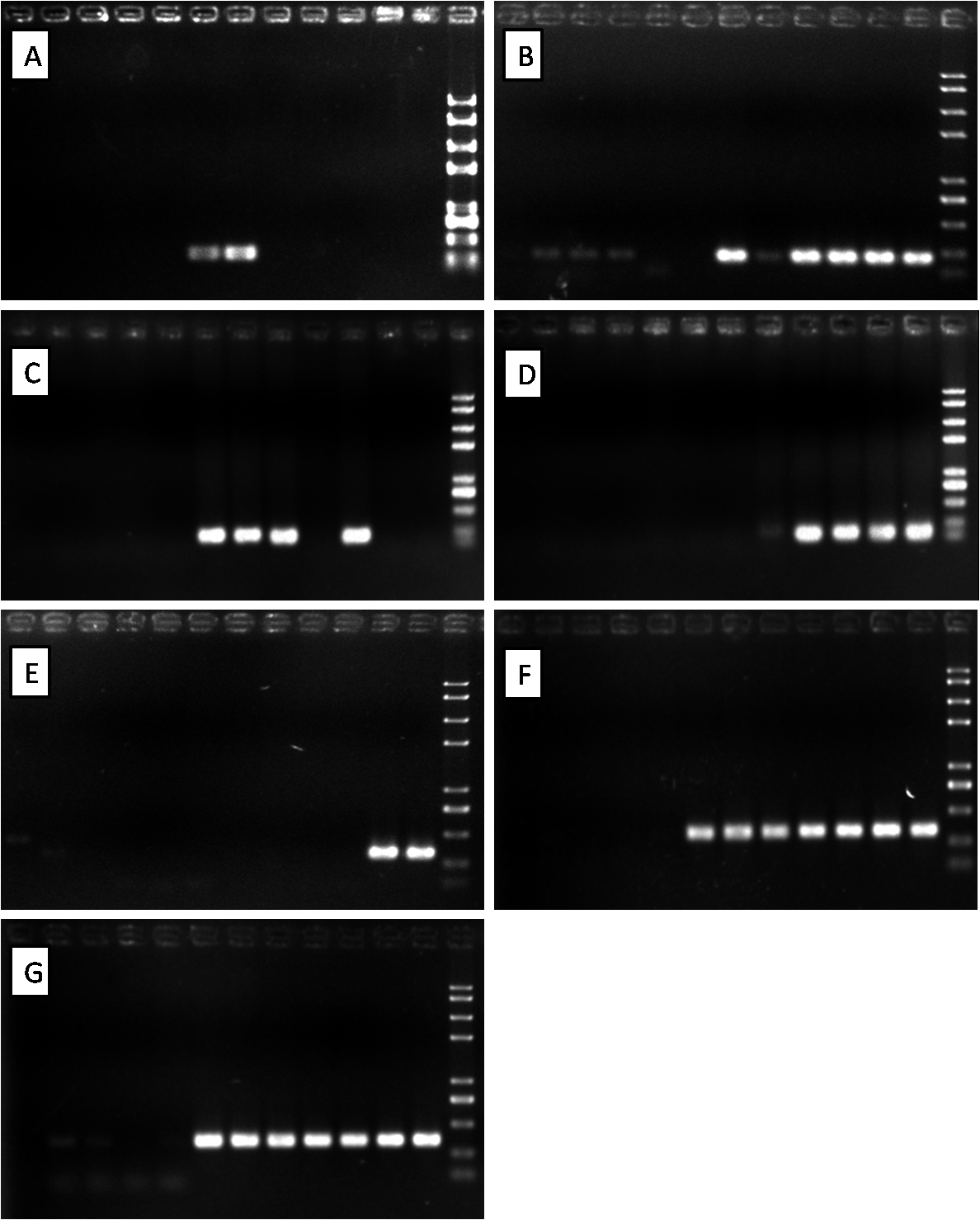


**Figure 2 Verification of the overlaps between clones in contig70**

In section A, B, C, D, E, F and G, the templates used in PCR, from left to right, are: empty vector, U13J12 (contig6), U03H01 (contig133), U03J10 (contig53), U03A03 (contig100), U15M14, U07D11, U13N13, U08F01, U08J02, U05L06 and U03J21. A: The pair primers were derived from BES of U07D11.f; B: The pair primers were derived from BES of U07D11.r; C: The pair primers were derived from BES of U08J02.f; D: The pair primers were derived from BES of U08J02.r; E: The pair primers were derived from BES of U03J21.f; F: The pair primers were derived from BES of U03J21.r; G: The pair primers were derived from BES of U15M14.r.

## D: Quality evaluation of contig50


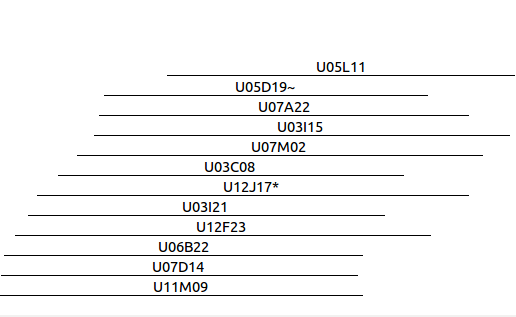


**Figure 1 Location of the clones in contig50**

* The clone U12J17 contains clone U05D19 in physical location in this contig.

**~** The clone U05D19 is contained by clone U12J17 in physical location in this contig.


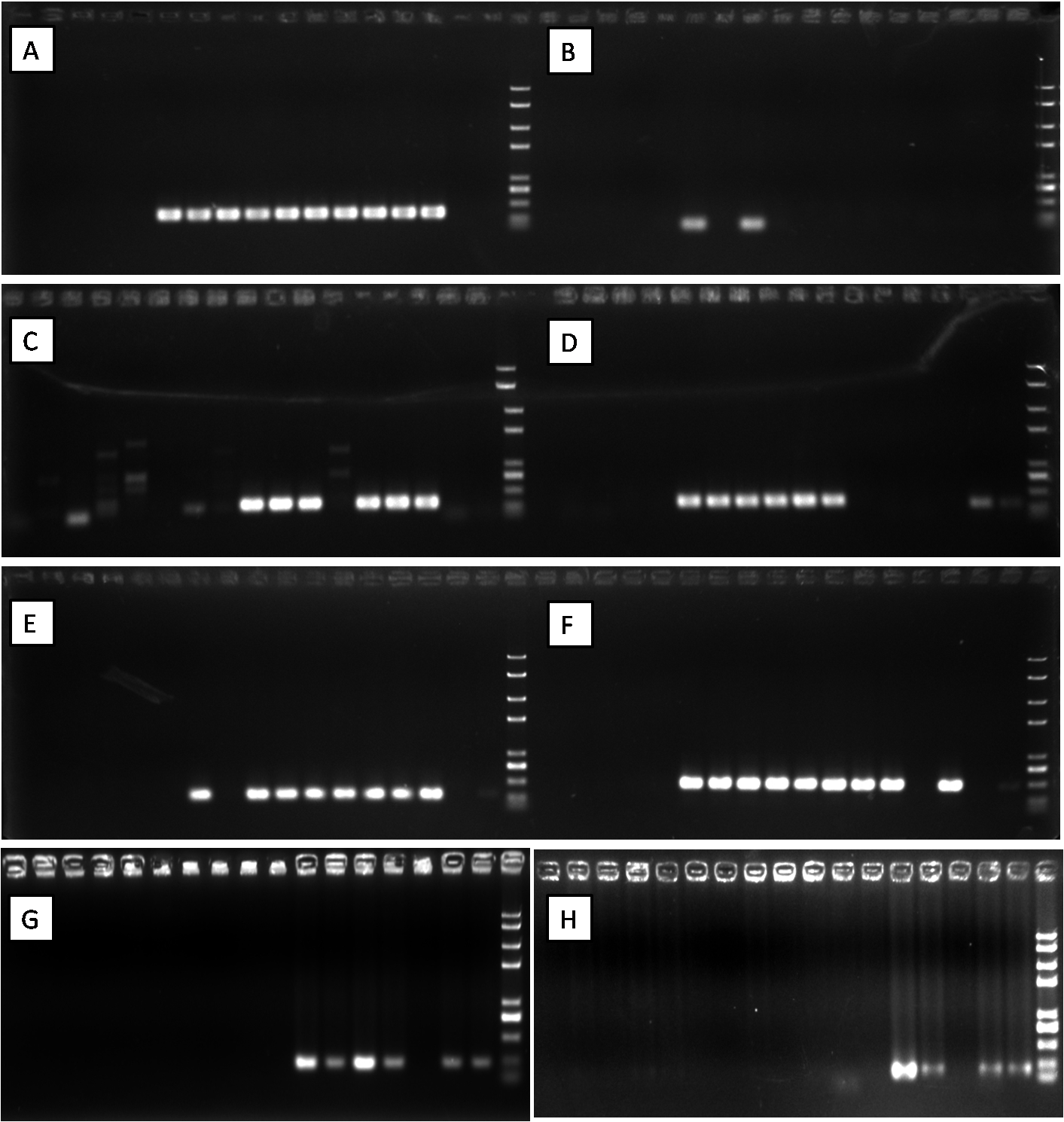


**Figure 2 Verification of the overlaps between clones in contig50**

In section A, B, C, D, E and F, the templates used in PCR, from left to right, are: empty vector, U13J12 (contig6), U03H01 (contig133), U03J10 (contig53), U03A03 (contig100), U11M09, U07D14, U06B22, U12F23, U03I21, U12J17, U03C08, U07M02, U03I15, U07A22, U05D19 and U05L11. A: The pair primers were derived from BES of U11M09.f; B: The pair primers were derived from BES of U11M09.r; C: The pair primers were derived from BES of U03I21.f; D: The pair primers were derived from BES of U03I21.r; E: The pair primers were derived from BES of U03C08.f; F: The pair primers were derived from BES of U03C08.r; G: The pair primers were derived from BES of U05D19.r; H: The pair primers were derived from BES of U05L11.f.

## E: Quality evaluation of contig36


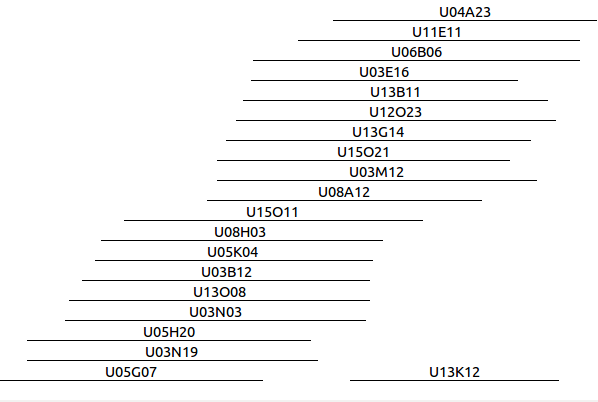


**Figure 1 Location of the clones in contig36**

**
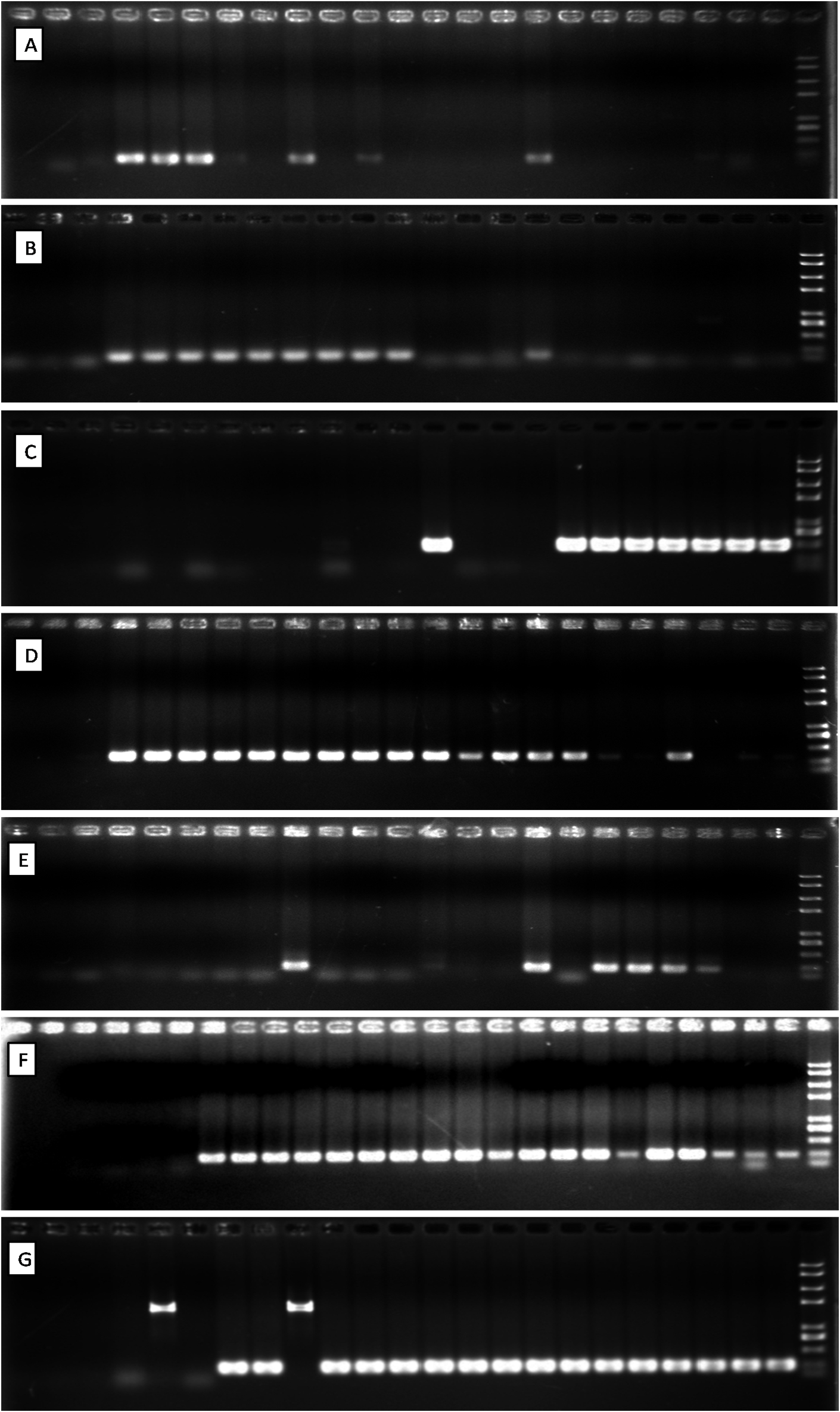
**

**Figure 2 Verification of the overlaps between clones in contig36**

In section A, B, C, D, E, F and G, the templates used in PCR, from left to right, are: empty vector, U03J10 (contig53), U03A03 (contig100), U05G07, U03N19, U05H20, U03N03, U13O08, U03B12, U05K04, U08H03, U15O11, U08A12, U03M12, U15O21, U13G14, U12O23, U13B11, U03E16, U06B06, U11E11, U04A23 and U13K12. A: The pair primers were derived from BES of U03N19.r; B: The pair primers were derived from BES of U15O11.f; C: The pair primers were derived from BES of U08A12.f; D: The pair primers were derived from BES of U13G14.f; E: The pair primers were derived from BES of U13G14.r; F: The pair primers were derived from BES of U03E16.f; G: The pair primers were derived from BES of U13K12.r.

## F: Quality evaluation of contig17


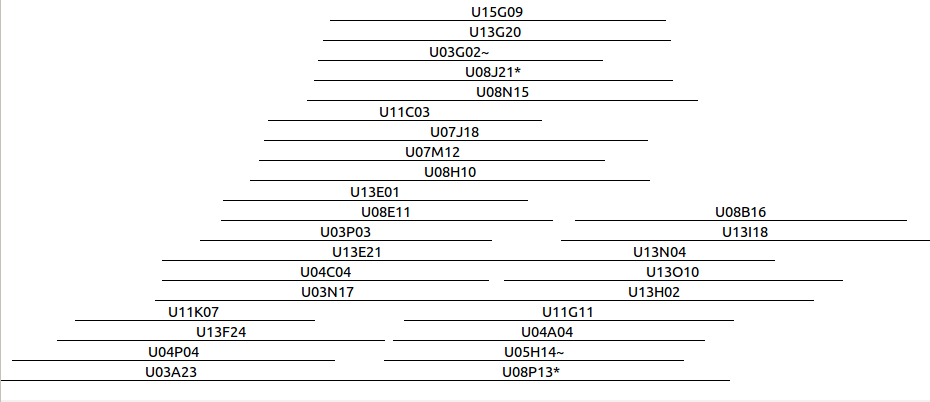


**Figure 1 Location of the clones in contig17**

* The clone U08J21 contains clone U03G02 in physical location in this contig.

**~** The clone U03G02 is contained by clone U08J21 in physical location in this contig.


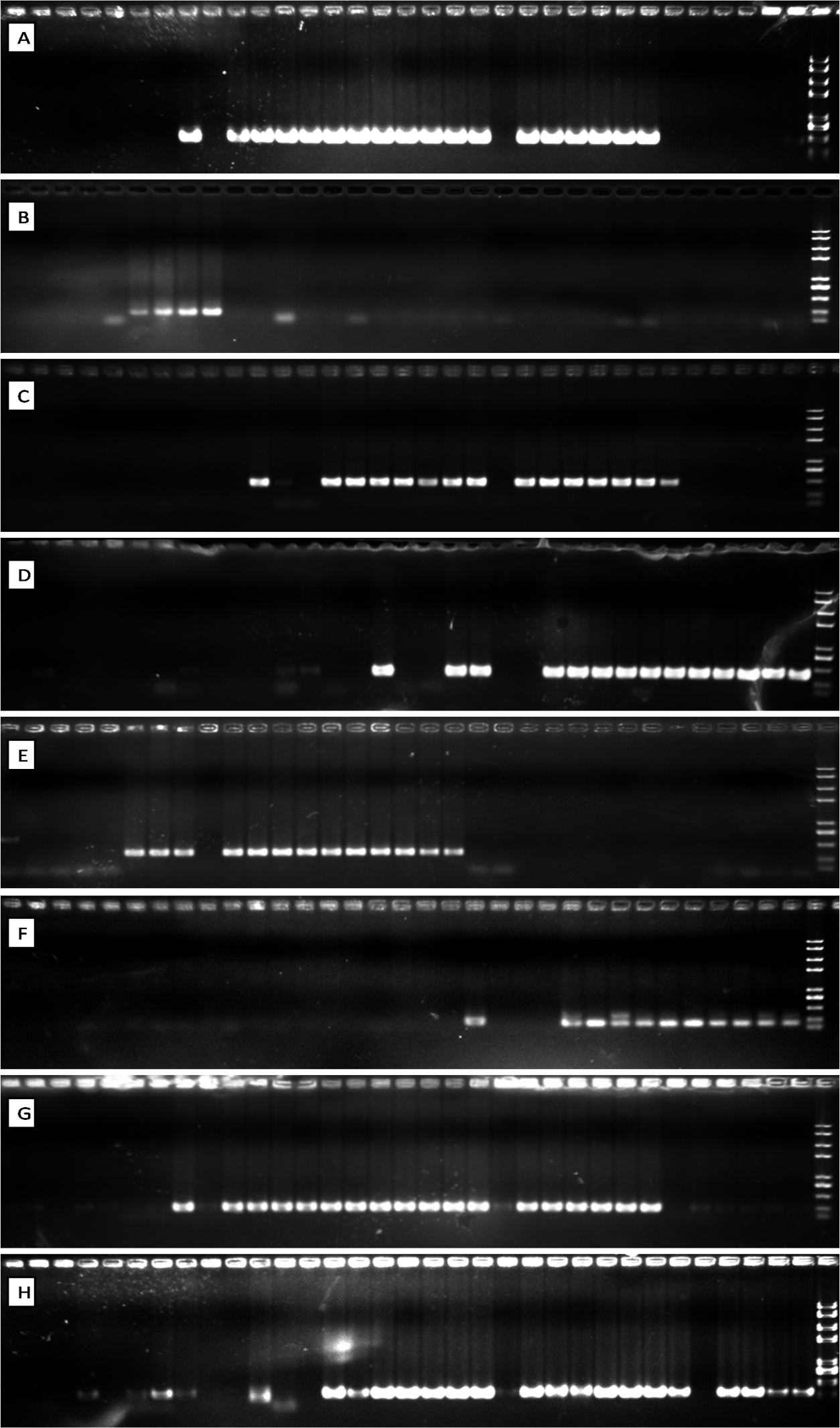


**Figure 2 Verification of the overlaps between clones in contig17**

In section A, B, C, D, E, F, G and H, the templates used in PCR, from left to right, are: empty vector, U13J12 (contig6), U03H01 (contig133), U03J10 (contig53), U03A03 (contig100), U03A23, U04P04, U13F24, U11K07, U03N17, U13E21, U04C04, U03P03, U08E11, U13E01, U08H10, U07M12, U07J18, U11C03, U08N15, U08J21, U03G02, U13G20, U15G09, U08P13, U05H14, U04A04, U11G11, U13H02, U13O10, U13N04, U13I18 and U08B16. A: The pair primers were derived from BES of U13F24.f; B: The pair primers were derived from BES of U13F24.r; C: The pair primers were derived from BES of U13E01.f; D: The pair primers were derived from BES of U11C03.f; E: The pair primers were derived from BES of U11C03.r; F: The pair primers were derived from BES of U08P13.f; G: The pair primers were derived from BES of U08P13.r; H: The pair primers were derived from BES of U13O10.f.

## G: primers used in this experiment

| Contig name | Primer name | Primer sequence | Product size |
| --- | --- | --- | --- |
| Contig184 | U08N11.f_F | CCCTGTTTCGCTGTTTGCAA | 181 |
|  | U08N11.f_R | TTGGAGACAGTGATGCGAGG |  |
|  | U08N11.r_F | CCCAAGATAACCAACGGCAC | 213 |
|  | U08N11.r_R | AAACGTGACGTCCGGGTATC |  |
|  | U15O06.r_F | TCTGTAGGAGTTGGCATGCG | 179 |
|  | U15O06.r_R | GGAGGTTGGGTTGACGAGAG |  |
| Contig155 | U12J05.f_F | CGTTGCCGTTTGTTATAGTTG | 371 |
|  | U12J05.f_R | CAGCAGACCAGGTGAAGC |  |
|  | U15M06.f_F | TGGTTCTTTGCGTTTGTC | 210 |
|  | U15M06.f_R | TAGCCACTTGCTGGTTTT |  |
|  | U15M06.r_F | CGTTGCCGTTTGTTATAGTTG | 373 |
|  | U15M06.r_R | CCCAGCAGACCAGGTGAA |  |
| Contig149 | U07I14.f_F | CTGGCAGTATGATTGTAGAAGGG | 197 |
|  | U07I14.f_R | CAAGGGTGAGATGGGTGTTTA |  |
|  | U15K23.f_F | GCATTCAATCGCACCTCACT | 137 |
|  | U15K23.f_R | GCCCGTTCGTTCAAATCTTC |  |
|  | U08B20.f_F | CAAGGAGTGGGCGTAGGT | 247 |
|  | U08B20.f_R | CGATAGCACTGCCGATTAC |  |
|  | U08B20.r_F | GCTAACTTGGTACGCACTTCTC | 246 |
|  | U08B20.r_R | CTGTCTCGCCAGGTGTCATT |  |
| Contig70 | U07D11.f_F | GGGTAGGTGCTAGGGCAAAC | 318 |
|  | U07D11.f_R | ACGGAGTAAAGCGAGGTGGA |  |
|  | U07D11.r_F | TGCAGCAGTTACCGAGACAT | 221 |
|  | U07D11.r_R | GGTGGGACCTTGGTGGAT |  |
|  | U08J02.f_F | CCTGCACAGGCTCTTGAC | 191 |
|  | U08J02.f_R | AGGATGTTGCTCGGCTTC |  |
|  | U08J02.r_F | TCACGTTCGTCAAGCTCCAT | 150 |
|  | U08J02.r_R | CGGTTGCTGCAAACAAGG |  |
|  | U03J21.f_F | ATGCCAACTTAAACATGCGAACT | 320 |
|  | U03J21.f_R | CCCTGAGCCCTGTCTCCCTA |  |
|  | U03J21.r_F | TATCACATCACGGCAGAAGAGG | 326 |
|  | U03J21.r_R | CCAGAAATCATACAAGGCACCC |  |
|  | U15M14.r_F | TTCGTCTGCTCCGCTTCCTG | 348 |
|  | U15M14.r_R | GGGTTGTAGATGACTCGGTTGAT |  |
| Contig50 | U11M09.f_F | CAAGGATGCACTTGGGAAGA | 363 |
|  | U11M09.f_R | AAGCGTAGCGGCGTATGG |  |
|  | U11M09.r_F | GGTTCCTCAGGTGCGTCAAT | 145 |
|  | U11M09.r_R | TTCTGGGCGACCCTCAAACA |  |
|  | U03I21.f_F | CCCCTGTTGTCGGGTGTTGC | 308 |
|  | U03I21.f_R | GGCTGACGTTCGGTTGCTTG |  |
|  | U03I21.r_F | TTGGGTTTGGTCTTTGTGCG | 319 |
|  | U03I21.r_R | TTCCTTCGTGGTCCTCGTCA |  |
|  | U03C08.f_F | ATTGAGCAGCGGGAGGTT | 307 |
|  | U03C08.f_R | CGTGGATGGCTTCGTTCTT |  |
|  | U03C08.r_F | ATGTAACCCGCCAACAGCAC | 466 |
|  | U03C08.r_R | CGACCGCAACCAAACAAGAT |  |
|  | U05D19.r-F | CATAGCCCGTACCATCCG | 241 |
|  | U05D19.r-R | ACCAGACCCGTTCCCTCA |  |
|  | U05L11.f-F | CTGGAGCCTTGTTCTGATGC | 176 |
|  | U05L11.f-R | GGACCCGAAGATGATTATGC |  |
| Contig36 | U03N19.r-F | CGACGAGGACAAGGAAGACT | 202 |
|  | U03N19.r-R | CAGGTTATCCAGGCGAGGT |  |
|  | U15O11.f-F | GCGGCTGCTATGTATGTATGTC | 115 |
|  | U15O11.f-R | AGAAAGCGATGTGATGGAGG |  |
|  | U08A12.f-F | GTGGGATGGAACGCTACTGG | 469 |
|  | U08A12.f-R | CCGATCTATCTCCTCGCTCTTTT |  |
|  | U13G14.f-F | TCAGCCTGGCACATCAAAGT | 325 |
|  | U13G14.f-R | TCAAGCAACGGACAATCAAG |  |
|  | U13G14.r-F | GTCGGGCATCGTTTCCTTCC | 214 |
|  | U13G14.r-R | TCTTCGTGCTTCCACCTCCA |  |
|  | U03E16.f-F | CGGAGTAGCCCTCGTCGTCT | 243 |
|  | U03E16.f-R | CTGGTGGTGCGTGGCGTAT |  |
|  | U13K12.r-F | TCAATCCTGTCGGCTTCCCTC | 240 |
|  | U13K12.r-R | CCCTTGTCGTCGGGCTTGTT |  |
| Contig17 | U13F24.f-F | GTTCAGGAGACGGTGACGC | 423 |
|  | U13F24.f-R | GGAAATAAGACAGCAAAGGAGG |  |
|  | U13F24.r-F | CCTCACCAACTCGCTGTCC | 160 |
|  | U13F24.r-R | AGCACCCATGCAAATCTCG |  |
|  | U13E01.f-F | GGAGAACCGCTGGTCAAGG | 427 |
|  | U13E01.f-R | CAGTCGCTGTCGCCAAGTC |  |
|  | U11C03.f-F | TCAAACCGCCATTGTTCCTT | 373 |
|  | U11C03.f-R | AGACCGTCGTCGCTTTCATC |  |
|  | U11C03.r-F | GGTCATGGCGGGCATCTTG | 318 |
|  | U11C03.r-R | GCGGGTTTCGTGGTTGTAGTG |  |
|  | U08P13.f-F | TCCATGCCAGTGTCAACG | 184r |
|  | U08P13.f-R | AGACTCAAACCCGCCAAA |  |
|  | U08P13.r-F | ACGGATTCGAGAATAGCC | 236 |
|  | U08P13.r-R | GATAGCAGCAGACCCTTG |  |
|  | U13O10.f-F | GAGGGCGAAGACGAGGCACA | 300 |
|  | U13O10.f-R | TGGCGGGATGGGTGTAGGTC |  |
